# Prevalence, probability, and outcomes of typhoidal/non-typhoidal *Salmonella* and malaria co-infection among febrile patients: a systematic review and meta-analysis

**DOI:** 10.1038/s41598-021-00611-0

**Published:** 2021-11-08

**Authors:** Polrat Wilairatana, Wanida Mala, Wiyada Kwanhian Klangbud, Kwuntida Uthaisar Kotepui, Pongruj Rattaprasert, Manas Kotepui

**Affiliations:** 1 Department of Clinical Tropical Medicine, Faculty of Tropical Medicine, Mahidol University, Bangkok, Thailand; 2 Medical Technology, School of Allied Health Sciences, Walailak University, Tha Sala, Nakhon Si Thammarat Thailand; 3 Department of Protozoology, Faculty of Tropical Medicine, Mahidol University, Bangkok, Thailand

**Keywords:** Epidemiology, Infectious diseases

## Abstract

The geographical overlaps of malaria parasites and *Salmonella* spp. can lead to co-infection of these two pathogens, especially in the tropics where malaria is endemic. Moreover, few literatures suggested that malaria infection was associated with *Salmonella* bacteremia. Therefore, this study quantified pooled prevalence of typhoidal/non-typhoidal *Salmonella* (NTS) and probability of typhoidal/NTS and malaria co-infection among febrile patients. The systematic review protocol was registered at PROSPERO (CRD42021252322). Studies on co-infection of typhoidal/NTS and malaria were searched in PubMed, Scopus, and Web of Science. The risk of bias of the included studies was assessed using the checklist for analytical cross-sectional studies developed by the Joanna Briggs Institute. Meta-analyses on the following criteria were performed: (1) pooled prevalence of typhoidal/NTS and malaria co-infection among febrile patients, (2) pooled prevalence of typhoidal/NTS among malaria patients, (3) pooled prevalence of malaria infections among patients with *Salmonella* spp. infection, and (4) probability of typhoidal/NTS and malaria co-infection among febrile patients. Additionally, the case fatality rate and mean difference of malarial parasitemia between typhoidal/NTS and malaria co-infection and *Plasmodium* monoinfection were also determined. The subgroup analyses of typhoidal/NTS, regions (Africa and Asia), countries, time (publication year), characteristics of participants, and diagnostic tests for identifying *Salmonella* spp. were also conducted. A sensitivity test was performed to determine the robustness of the study outcomes. Publication bias among the included studies was evaluated using the funnel plot and Egger’s test. All analyses were performed using Stata version 15 (StataCorp LLC, Texas, USA) with a p-value < 0.05 indicating statistical significance. Eighty-one studies that met the eligibility criteria were included in the analyses. Of the 73,775 study participants, 4523 had typhoidal/NTS and malaria co-infections. The pooled prevalence rates of typhoidal/NTS and malaria co-infection among febrile patients were 14% (95% confidence interval [CI], 9–19%; I^2^, 99.4%; 2971/17,720 cases) and 1% (95% CI 1–1%; I^2^, 89.9%; 252/29,081 cases) using the Widal test and culture methods for identifying *Salmonella* spp., respectively. The pooled prevalence rates of typhoidal/NTS infection among patients with malaria were 31% (95% CI 23–39%; I^2^, 99.5%; 3202/19,208 cases) and 3% (95% CI 2–3%; I^2^, 86.8%; 407/40,426 cases) using the Widal test and culture methods for identifying *Salmonella* spp., respectively. The pooled prevalence rates of malaria infection among patients with typhoidal/NTS were 17% (95% CI 6–29%; I^2^, 33.3%; 13/75 cases) and 43% (95% CI 32–53%; I^2^, 89.1%; 287/736 cases), respectively. Malaria infection was associated with typhoidal/NTS in children aged < 15 years (p < 0.0001; odds ratio, 0.36; 95% CI 0.23–0.58; I^2^, 73.9%; 3188/43,212 cases). The case fatality rate in patients with malaria and NTS co-infections was 16% (95% CI 9–24%; I^2^, 89.1%; 18/103 cases). From the view of the present study, the inappropriate use of the Widal test for *Salmonella* spp. diagnosis can overestimate the prevalence of typhoidal/NTS and malaria co-infections*.* Malaria infection associated with typhoidal/NTS in children and the high case fatality rates among few patients with co-infections were highlighted. Future prospective longitudinal studies using the appropriate and confirmatory dsiagnosis for *Salmonella* spp. infections are highly recommended to ensure the real prevalence of co-infection and highlight the outcome of co-infection for providing adequate treatment in febrile patients who live in areas where malaria is endemic, such as tropical Africa and India.

## Introduction

Malaria is a major public health problem in tropical and subtropical countries. The World Health Organization (WHO) reported 229 million cases of malaria and 409,000 deaths in 2019^1^. Most of these cases (51% globally) were found in Nigeria (27%), the Democratic Republic of the Congo (12%), Uganda (5%), Mozambique (4%), and Niger (3%), whereas 51% of malaria deaths occurred in Nigeria (23%), the Democratic Republic of the Congo (11%), the United Republic of Tanzania (5%), Mozambique (4%), Niger (4%), and Burkina Faso (4%)^1^. Although the incidence of malaria declined from 2000 to 2019, its diagnosis among febrile patients in malaria-endemic settings remains challenging; malaria and typhoid and non-typhoid fever are co-endemic and have similar clinical signs and symptoms.

*Salmonella* species are Gram-negative bacilli members of Enterobacteriaceae and associated with human infection^2,3^. *Salmonella* is comprised of two major species, namely, *Salmonella enterica* and *Salmonella bongori*. *S. enterica* are classified into six serotypes, which are differentiated based on their antigenicity^3^. Several *Salmonella enterica* serotypes cause typhoid fever. *S. typhi* and *S. paratyphi*, collectively referred to as typhoidal *Salmonella*, are the most common species that cause enteric fever (typhoid fever and paratyphoid fever). While both fevers share clinical symptoms, paratyphoid fever tends to be more benign^4,5^. Paratyphoid fever is most commonly acquired by ingesting contaminated food or water^5^. Humans are key reservoir hosts for typhoidal *Salmonella* and contribute to disease transmission and dissemination^5^. Typhoid fever is characterized by gastroenteritis and presents with nonspecific clinical symptoms, such as high fever, fatigue, headache, malaise, abdominal pain, nausea, vomiting, constipation, and diarrhea^5,6^. These symptoms are indistinguishable from other causes of fever, such as malaria^5^. The complications of typhoid fever include septicemia, meningitidis, and immunological symptoms^7^. Typhoid fever may be mild or severe, and complications may contribute to typhoid-related deaths^7,8^. Non-typhoid fever is a febrile illness caused by non-typhoidal *Salmonella* (NTS), including *S. enteritidis* and *S. typhimurium*^9^. NTS infections most often cause mild gastroenteritis, which is usually self-limiting^7^. Recently, NTS infections have been associated with septicemia and high mortality rates in immunocompromised patients in sub-Saharan Africa^10^.

The *Salmonella* infection rate is high in low- and middle-income countries with > 100 per 100,000 infected people annually^11,12^. Typhoid fever is an important cause of morbidity and mortality worldwide, with an estimated 16–33 million cases and 500,000 to 600,000 deaths annually^13^. Typhoid is endemic in developing countries, especially Africa, whereas developed countries have a much lower incidence. The majority of patients in developed countries are travelers returning from endemic areas^14^. In developing countries, especially in Southeast Asia and Africa, NTS is endemic and is a global burden, contrary to typhoidal *Salmonella*^12,14^. Poor water quality, poor handwashing habits, and consumption of untreated drinking water or unsafe food are the main causes of typhoid fever^15^. Therefore, people with low socioeconomic status and poor or improper hygiene have high risk of fecal–oral enteric infections, including typhoidal and NTS. The most recent meta-analysis revealed that household behaviors, including poor hygiene and consumption of unsafe food and untreated water, increase the risk of typhoid transmission^16^. Among children in Africa, NTS is a leading cause of bacteremia, whereas typhoid fever has a relatively low burden^17–19^. Another study demonstrated that typhoid fever is more common in older children with a period of fever, whereas non-typhoidal bacteremia frequently develops in younger children of poorly educated women or women with low socioeconomic status^20^.

For the diagnosis of typhoidal *Salmonella* infection, especially from blood, bacteriological culture is the gold standard^21^. The sensitivity of the culture method depends on the blood volume, antibiotic treatment, affected individual, disease duration, and presence of bacteremia^22^. Blood cultures have a sensitivity of 40–80%^9,23^. Moreover, they are most sensitive in the first week of infection as circulating bacterial concentrations peak at that time^23^. Stool and rectal swab cultures have lower sensitivity than blood cultures^24^. However, sensitivity can be enhanced by culturing from three specimens or performing multiple cultures from a single stool specimen^24^. Culture methods are less frequently employed in developing countries because of the high cost and requirements for good laboratory facilities and highly trained professionals^7,25^. Serological diagnoses of infections are conducted using the Widal test. This test measures the antibody titers specific for *Salmonella* O (somatic) and H (flagella) antigens^7^. The Widal test is widely used in numerous countries where trained technicians and laboratory facilities are limited^7,25^. Other useful methods for the diagnosis of *Salmonella* infection include enzyme-linked immunosorbent assay (ELISA), which detect IgM and IgG antibodies against *Salmonella* surface molecules, and molecular methods, such as nested multiplex polymerase chain reaction (PCR) and real-time PCR, which target *Salmonella* virulence genes. The real-time PCR test is highly specific and sensitive and has faster turnaround times than culture methods^7,26^.

The geographical overlaps of malarial parasites and typhoidal/NTS can lead to co-infection of these two pathogens, especially in the tropics where malaria is endemic. The overlap in the clinical symptoms of malaria and non-malaria febrile illness or co-infection of these two pathogens may lead to the misdiagnosis of one disease. Previous studies conducted in Africa demonstrated that bacteremia caused by NTS was associated with malaria parasitemia^17,27,28^, recent malaria^29^, anemia^29^, severe malarial anemia^30^, jaundice, and hypoglycemia^20^. Previous studies also demonstrated that NTS infection is associated with more severe anemia and malaria compared with typhoidal *Salmonella* or other bacteremia infections^17,29,31^. Another study demonstrated that NTS infections were associated with previous antimalarial treatment and malarial complications (severe anemia, jaundice, and hypoglycemia). Furthermore, a systematic review demonstrated a higher case fatality rate in children who were co-infected with NTS compared with those infected with malaria alone; however, the study had limitations on high heterogeneity between studies, inclusion of recent malaria infection, use of antigen-based rapid diagnostic tests (RDTs), study design, quality of microbiological data, and publication bias, making the meta-analysis potentially misleading^32^. To the best of our knowledge, meta-analyses determining the association between malaria and typhoid/non-typhoid fever have not been well conducted, and information is not updated. Therefore, the present study aimed to quantify the pooled prevalence, probability, and outcome of typhoidal/NTS and malaria co-infection among febrile patients who were suspected of having these two diseases.

## Methods

### Protocol and registration

The protocol of systematic review was registered at PROSPERO (CRD42021252322) and conducted according to the Preferred Reporting Items for Systematic Reviews and Meta-analyses (PRISMA) statement^33^.

### Search strategy

Potentially relevant articles in PubMed, Web of Science, and Scopus were searched using the combined search terms presented in Supplementary Table S1. The relevant search terms were retrieved from Medical Subject Headings to ensure the inclusion of all relevant studies. The searches were conducted from inception to April 27, 2021. Searches were limited to the English language, but the year of publication was not limited. Additional searches were performed by reviewing the reference lists of the included studies and Google Scholar to ensure that all potentially relevant studies were included in the meta-analysis.

### Eligibility criteria

Observational studies in the English language that reported concurrent malaria and typhoidal/NTS infection were included in the study. Studies reporting data that could not be extracted, case–control studies, experimental studies, animal studies, case reports, and case series were excluded.

### Study selection and data extraction

Potentially relevant articles were selected by two authors (MK, WM) using the eligibility criteria. First, the duplicates from the three databases were removed. Second, the remaining studies were screened for titles and abstracts, and any non-related studies were excluded. Third, the full texts of the remaining studies were examined, and any non-related studies were excluded with reasons. Then, the remaining studies were included in the systematic review and meta-analysis. Any disagreement on the study selection between the two authors was resolved by reaching a consensus after the discussion. Data extraction was performed by two authors (MK, WM) using the pilot standardized datasheets. The following information was obtained from each study: first author names, publication year, study sites (country and region), year the study was conducted, study design, characteristics of participants including age and sex, number of co-infections, number of malaria cases, number of typhoid/non-typhoid cases, number of case fatality in co-infection and *Plasmodium* monoinfection, diagnostic test for malaria, and diagnostic test for typhoid (best diagnostic test). Any disagreement on data extraction between the two authors was resolved by a third author (PW) for the final decision.

### Risk of bias

The risk of bias of the included studies was evaluated using the checklist for analytical cross-sectional studies developed by the Joanna Briggs Institute^34^. The checklist is comprised of eight categories (yes/no/unclear/not applicable answers) based on the design, conduct, and analysis. Studies with yes answers in all eight categories were considered to have low risk of bias (high quality), whereas those that complied with four to six categories were considered to have a moderate risk of bias (moderate quality). Any study that complied with less than four categories was considered to have a high risk of bias (low quality) and thus excluded from the present study. The risk of bias was evaluated by two authors (MK, WM). If the two authors disagreed on the risk of bias assessment, a third author (PW) was responsible for the final decision.

### Outcomes

The outcomes of this study were as follows: (1) pooled prevalence of typhoidal/NTS and malaria co-infection among febrile patients, (2) pooled prevalence of typhoidal/NTS infection among patients with malaria, (3) pooled prevalence of malaria infection among patients with *Salmonella* spp. infection, (4) comparison of typhoidal/NTS infection among patients with severe and non-severe malaria, (5) association between malaria and typhoidal/NTS infections, (6) case fatality rate among patients with typhoidal/NTS and malaria co-infection, and (7) difference in mean parasitemia level between patients with typhoidal/NTS and malaria co-infection and those with *Plasmodium* spp. monoinfection.

### Data synthesis

The pooled prevalence rate of typhoidal/NTS and malaria co-infection among febrile patients, typhoidal/NTS infection rate among patients with malaria, malaria infection rate among patients with *Salmonella* spp. infection, case fatality rate among patients with typhoidal/NTS and malaria co-infection, and comparison of typhoidal/NTS infection rates among patients with severe and non-severe malaria were estimated using random-effect models, assuming heterogeneity of the included studies. The results of the individual studies are presented in the forest plots as the point estimates (prevalence in percentage) and 95% confidence interval (CI). The association between *Plasmodium* spp. and *Salmonella* spp. infections was determined using the random-effects model and expressed as odds ratio with 95% CI. The difference in mean parasitemia level between patients with typhoidal/NTS and malaria co-infection and *Plasmodium* spp. monoinfection was estimated using the random-effects model and expressed as weighted mean difference (WMD) with 95% CI. The heterogeneity among the included studies was assessed using Cochran’s Q and I^2^ statistics. Cochran’s Q < 0.05 or I^2^ > 50% indicated substantial heterogeneity among the included studies. If no substantial heterogeneity existed, the fixed-effects model was employed to estimate the effect size (pooled prevalence or pooled odds ratio). The subgroup analysis of typhoidal/NTS, regions (Africa and Asia), countries, time (publication year), characteristics of participants, and diagnostic tests for identifying *Salmonella* spp. were conducted to explore the source(s) of heterogeneity among the overall effect estimate. Sensitivity analyses of the probability of *Plasmodium* spp. and *Salmonella* spp. co-infection were performed using the random- and fixed-effects models after excluding outliers.

### Publication bias

Publication bias among the included studies was evaluated using a funnel plot between the effect size (ES) and standard error of the ES (seES). A funnel plot with asymmetrical distribution indicated publication bias. Egger’s test was employed if the funnel plot asymmetry was caused by the small study effect. A contour-enhanced funnel plot was also utilized to find the possible causes of funnel plot asymmetry among the included studies. The significance of contour-enhanced funnel plots (p < 0.01) indicated that the cause of funnel plot asymmetry might be more likely other factors such as heterogeneity, selection bias, and quality of the included studies than publication bias.

## Results

### Search results

**Figure 1:**
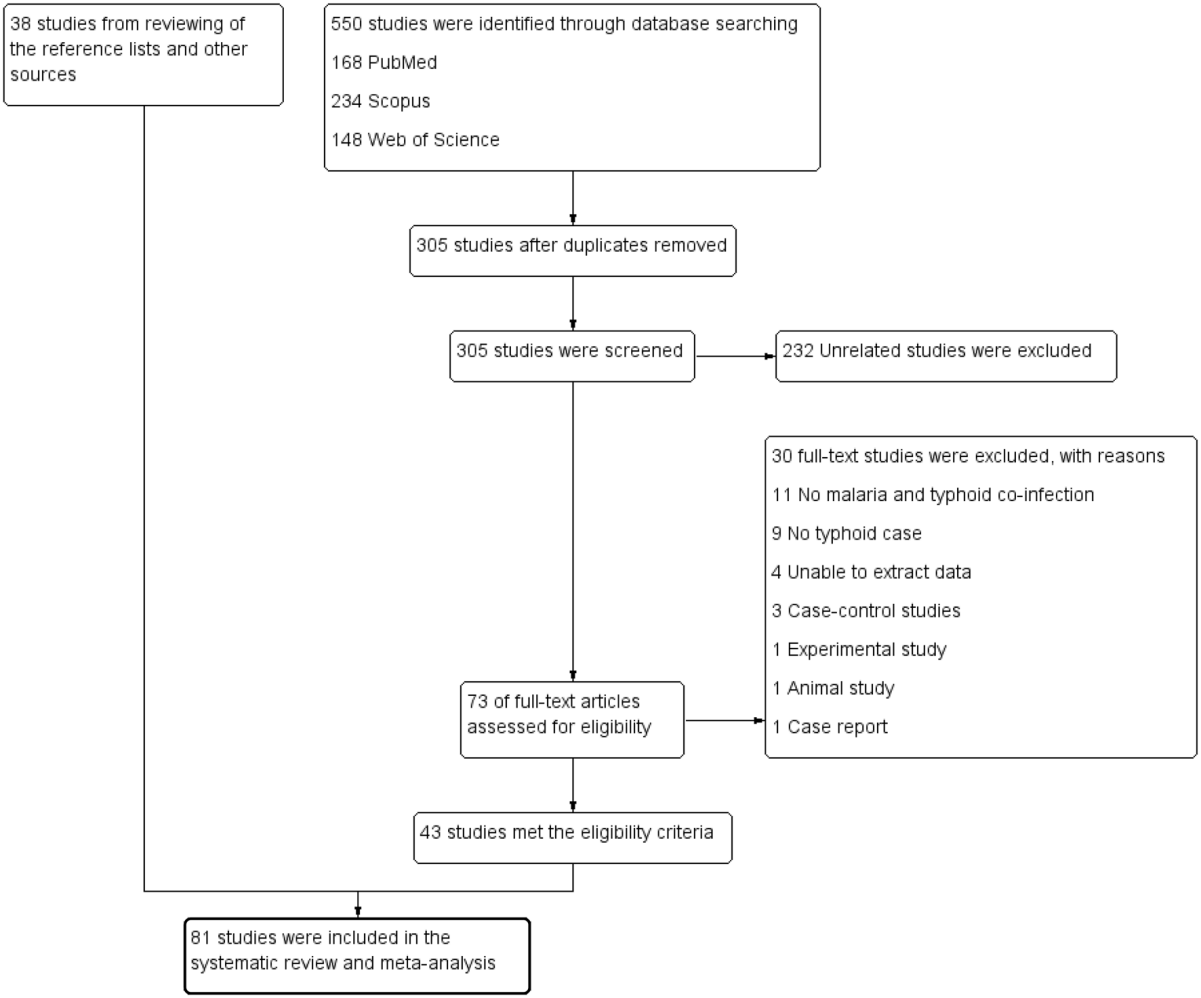
Study flow diagram.

A total of 550 studies were retrieved from the three databases (168 from PubMed, 234 from Scopus, and 148 from Web of Science). After removal of 245 duplicated studies, the titles and abstracts of 305 studies were screened. After excluding 232 unrelated studies, 73 were retained for full-text examination. A total of 30 studies were excluded for the following reasons: 11 studies had no malaria and typhoid co-infection cases, 9 studies had no typhoid cases, data could not be extracted from 4 studies, 3 were case–control studies, 1 was an experimental study, 1 was an animal study, and 1 study was a case report. Finally, 43 studies^17,20,30,35–74^ were included. Thirty-eight studies^27–29,31,75–108^ from additional searches of reference lists and Google Scholar were included. Thus, 81 studies met the eligibility criteria and thus included in the qualitative and quantitative analyses (Fig. 1).

### Characteristics and quality of the included studies

The characteristics of the included studies are presented in Table 1. A total of 76 studies were cross-sectional or retrospective studies, whereas 5 were prospective studies. All studies were published between 1987 and 2021. In Africa, 61 studies (75.3%) were conducted; in Asia, 19 studies (23.4%); and in Europe, 1 study. The African studies were conducted in Nigeria (30/61, 49.2%)^36–38,42,47,48,50,51,57,59,61,64,75,77,82–84,89–91,94–101,103,107^, Cameroon (5/61, 8.2%)^35,40,73,92,93^, Ghana (5/61, 8.2%)^41,53,56,76,85^, Kenya (5/61, 6.2%)^28,29,60,72,80^, Tanzania (4/61, 6.56%)^20,39,44,46^, Malawi^17,27,30^, Burkina Faso^54,86^, Mozambique^79^, Sierra Leone^87^, the Democratic Republic of the Congo^49^, Ethiopia^45^, Gabon^55^, and Gambia^31^, and one study was conducted in Burkina Faso, Ethiopia, Ghana, Guinea-Bissau, Kenya, Madagascar, Senegal, South Africa, Sudan, and Tanzania^62^. The Asian studies were conducted in India (12/19, 63.2%)^43,66–68,71,74,81,88,104–106,108^, Pakistan (4/19, 21.1%)^52,65,70,102^, Myanmar^58,78^, and Vietnam^63^. One study was conducted in Sweden^69^.

**Table 1:** Characteristics of the included studies.

Author	Study site	Year conducted	Study design	Participants	Age	Sex (M:F)	All co-infection	*Salmonella* spp. with typhoid	*Salmonella* spp. with non-typhoid	All malaria cases	Malaria without typhoid	Typhoid without malaria	Test for malaria	Test for typhoid
Abah et al. (2019)^75^	Nigeria	2016	Cross-sectional study	500 Febrile patients	1–60 years	244:256	115	115	0	278	163	85	Microscopy	Widal test
Achonduh-Atijegbe et al. (2016)^35^	Cameroon	2014	Cross-sectional study	315 Febrile children (6 months–15 years)	5.8 years (± 3.8)	157:158	14	14		193	179	*S. typhi* and *S. paratyphi* (14)	Microscopy, RDT, PCR	Rapid diagnostic test
Afoakwah et al. (2011)^76^	Ghana	NS	Cross-sectional study	129 Patients clinically diagnosed as having malaria	5–83 years	0.58125	6	6		24	18	26	RDT	Widal test
Agwu et al. (2009)^36^	Nigeria	2003–2004	Cross-sectional study	560 Febrile known HIV/AIDS (239 male and 321 female) patients	< 10 years (30), 11–20 (86), 21–30 (252), 31–40 (183), 41–50 (7), > 50 (2)	239:321	117	117		418	*P. falciparum* (301)	*S. typhi* (73)	Microscopy	Widal test
Akinyemi et al. (2007)^37^	Nigeria	2004–2005	Cross-sectional study	235 Febrile patients	0–5 years (29), 6–15 (31), 16–30 (22), 31–45 (15), > 46 (10)		16	16	0	107	91	26	Microscopy	Blood culture
Akinyemi et al. (2015)^38^	Nigeria	2010–2011	Cross-sectional study	135 Febrile patients	NS	NS	4	4		9	5	*S. typhi* (22), *S. paratyphi* (7)	Microscopy	Blood culture
Alhassan et al. (2012)^77^	Nigeria	NS	Cross-sectional study	300 Febrile patients	0 to > 60 years	143:157	4	4		51	47	NS	Microscopy	Blood culture
Ali et al. (2020)^39^	Tanzania	2015	Cross-sectional study	149 Febrile patients	Mean, 22 years; range, 1–70	62:87	1	1		7	6	0	Molecular method	Molecular method
Ammah et al. (1999)^40^	Cameroon	1997–1998	Cross-sectional study	200 Febrile patients	Mean, 28 years (± 20.1); range, 4–75	88:112	103	38	65	115	12	*S. typhi* (10), *S. paratyphi* (5), *S. typhimurium* (4)	Microscopy	Widal test
Anabire et al. (2018)^41^	Ghana	2015	Cross-sectional study	150 Febrile children	Median, 3 years (IQR, 2–8 years)	77:73	9	9		85	76; median age, 4.0 (2.0–8.0); anemia (55/76); thrombocytopenia (47/76); leukopenia (2/76); CBC	*S. typhi* (20): median age, 6.5 (3.0–11.0); anemia (12/20); thrombocytopenia (2/20); leukopenia (0); CBC	Microscopy, RDT	Widal test
Anjorin et al. (2020)^42^	Nigeria	2016–2018	Cross-sectional study	182 Pregnant women having influenza-like illness	Mean, 29.3 years; range, 18–45		34	34		34			NS	Rapid diagnostic test
Aung et al. (2018)^78^	Myanmar	2016–2017	Prospective study	20 Patients with *P. falciparum*	> 16 years	19:01	1	1		20	19	NS	Microscopy, RDT	Blood culture
Bassat et al. (2009)^79^	Mozambique	2003–2007	Retrospective study	1404 Children with severe malaria	< 5 years	NS	12	0	12	1404	1382	NS	Microscopy	Blood culture
Berkley et al. (1999)^80^	Kenya	1993–1996	Prospective study	783 Children with severe malaria	NS	396:387	6	0	6	783	777	NS	Microscopy	Blood culture
Bhalla et al. (2019)^43^	India	2018	Cross-sectional study	607 Patients with dengue, malaria, leptospirosis, typhoid, and rickettsia diseases	NS	Positive cases (male, 383:224)	1	1		372	371	45	Microscopy	Widal test
Bhattacharya et al. (2013)^81^	India	2004	Prospective study	3371 Febrile patients	Mean, 24.7 years	1730 :1641	2	2	0	93	91	159	Microscopy	Blood culture
Biggs et al. (2014)^44^	Tanzania	2006–2007	Cross-sectional study	3639 Febrile children	Median 1.57 years (0.2–13.0)	1970: 1669	53		53	2195	2142	*S. typhi* (11), non-typhoidal Salmonella (109)	Microscopy, RDT	Blood culture
Birhanie et al. (2014)^45^	Ethiopia	2013	Cross-sectional Study	200 Febrile patients	24.24 ± 13.4, range 2 to > 46	120:80	13	13		73	60	25	Microscopy	Widal test
Brent et al. (2006)^29^	Kenya	1998–2002	Prospective study	166 Non-typhoidal Salmonella	Median 15 months (8–27)		54	0	54	54	0	112	ELISA	Blood culture
Bronzan et al. (2007)^30^	Malawi	1996–2005	Cross-sectional study	1388 Severe malaria with bacteriamia	Children > 6 months		37		37	1388	1351	NS	Microscopy	Blood culture
Chipwaza et al. (2015)^46^	Tanzania	2013	Cross-sectional study	370 Febrile patients	2–13 years	189:181	13	13		98	85	38	Microscopy	Widal test
Chukwuma et al. (2014)^82^	Nigeria	2012–2013	Cross-sectional study	350 Pregnant women			5	5	0	10	5	1	Microscopy	Stool culture
Edet et al. (2016)^83^	Nigeria	2014–2015	Cross-sectional study	100 Febrile patients	10–80 years	43:57:00	11	11	0	41	30	0	Microscopy	Blood culture
Ekesiobi et al. (2017)^84^	Nigeria	NS	Cross-sectional study	256 Febrile patients	1 to > 35 years	128:128	29	25	4	202	173	9	Microscopy	Stool culture
Enabulele et al. (2016)^47^	Nigeria	NS	Cross-sectional study	271 Febrile patients	> 18 years	NS	5	5		193	188	24	Microscopy	Widal test
Evans et al. (2004)^85^	Ghana	NS	Cross-sectional study	23 Children with severe malaria	NS	NS	10	0	10	23	13	19	Microscopy	Blood culture
Eze et al. (2011)^48^	Nigeria	NS	Cross-sectional study	25 Malaria cases	NS	NS	3	3		25	22	0	NS	Widal tes t
Falay et al. (2016)^49^	Democratic Republic of the Congo	2012	Cross-sectional study	16 *S. typhi* positive, 107 Non-typhoidal *Salmonella*	*S. typhi* positive (median 96 (48–123) months), non-typhoidal *Salmonella* (median 24 (12–36))	*S. typhi* positive (7:9), non-typhoidal *Salmonell*a (63:45)	63	5	58		0	*S. typhi* positive (11), non-typhoidal *Salmonella* (59)	Microscopy, RDT	Blood culture
Graham et al. (2000)^17^	Malawi	1996–1998	Cross-sectional study	219 Non-typhoidal *Salmonella*	> 6 months	NS	82		82		0	144	Microscopy	Blood culture
Ibrahim et al. (2019)_86_	Burkina Faso	2014	Cross-sectional study	283 Malaria cases	Median 18 (0–85 years)	140:143	91	91	0	283	192	NS	Microscopy	Widal test
Igbeneghu et al. (2009)^50^	Nigeria	NS	Cross-sectional study	258 Febrile Patients	NS	NS	1	1		161	160	1	Microscopy	Blood culture
Igharo et al. (2012)^51^	Nigeria	NS	Cross-sectional study	234 Febrile patients	NS	113:121	43	43		88	45	130	Microscopy	Widal test
Jalani et al. (2019)^52^	Pakistan	2017	Cross-sectional study	144 Febrile patients	1–10 (75), 11–20 (35), > 20 (34)	74:70	9	9		20	11	86	Microscopy	Widal test
Kargbo et al. (2014)^87^	Sierra Leone	2013–2014	Cross-sectional study	11,069 Febrile patients	5–70 years	5245: 5824	2101	2101	0	8849	6748	554	Microscopy	Widal test
Katiyar et al. (2020)^88^	India	2018	Cross-sectional study	780 Malaria cases	0–80 years	425:355	122	122	0	780	658	NS	Microscopy, RDT	Widal test
Krumkamp et al. (2016)^53^	Ghana	2007–2012	Cross-sectional study	6746 Febrile patients	< 15 years		33		33	2563	2530	Non-typhoidal *Salmonella* (160), *S. typhi* (93)	Microscopy	Blood culture
Mabey et al. (1987)^31^	Gambia	1979–1984	Cross-sectional study	116 Patients with typhoidal/non-typhoidal *Samonella*			35	5	30	35	NS	81	Microscopy	Blood culture
Maltha el al. (2014)^54^	Burkina Faso	2012–2013	Cross-sectional study	711 Severe malaria	Median 19 (10–36)	393:318	33	12	21	711	678	0	Microscopy, RDT	Blood culture
Mbuh et al. (2003)^89^	Nigeria	1996	Cross-sectional study	218 Febrile patients	2–59 years	118:100	1	1	0	60	59	0	Microscopy	Blood culture
Mike et al. (2017)^90^	Nigeria	2015	Retrospective study	627 Febrile patients	1–75 years	375:252	136	136	0	233	97	49	Microscopy	Widal test
Mohammed et al. (2020)^91^	Nigeria	2020	Cross-sectional study	429 Pregnant women	21–30 years	429	12	12	0	123	111	33	Microscopy, RDT	Rapid diagnostic test
Mourembou et al. (2016)^55^	Gabon	NS	Cross-sectional study	410 Febrile patients	< 16 years	212:198	3			323	320	0	Molecular method	Molecular method
Mtove et al. (2010)^20^	Tanzania	2008–2009	Cross-sectional study	156 Children with pathogenic bacteriamia	2 months to 14 years		34	3	31		0	*S. typhi* (11), non-*S.* Typhi (14)	Microscopy, RDT	Blood culture
Ndip et al. (2015)^92^	Cameroon	2010	Cross-sectional study	206 Febrile patients	4–80 years		12	12	0	186	174	14	Microscopy	Stool culture
Nielsen et al. (2015)^56^	Ghana	2007–2011	Cross-sectional study	771 Malaria cases	< 15 years	1049:866	21		21	771	750	0	Microscopy	Blood culture
Njolle et al. (2020)^93^	Cameroon	2015	Cross-sectional study	160 Febrile patients	18 months–60 years	81:79	12	9	3	31	19	Typhoid (55), non-typhoidal *Salmonella* (24)	Microscopy	Stool culture
Nwabueze et al. (2013)^94^	Nigeria	NS	Cross-sectional study	700 Pregnant women	NS	NS	236	236	0	512	276	NS	Microscopy	Widal test
Nwuzo et al. (2009)^57^	Nigeria	2007	Cross-sectional study	250 Febrile patients	0–70 years	123:127	14	14		33	19	*S. typhi* (39)	RDT	Blood culture
Nyein et al. (2016)^58^	Myanmar	2014–2015	Cross-sectional study	67 Adults with *P. falciparum*	Adults	NS	4	3	1	67	63	0	Microscopy, RDT	Blood culture
Odikamnoro et al. (2018)^59^	Nigeria	NS	Cross-sectional study	350 Febrile patients	All age groups	164:186	127	127		190	63	46	Microscopy	Widal test
Ohanu et al. (2003)^95^	Nigeria	1997–1998	Cross-sectional study	270 Febrile patients	15–59 years	130:140	16	16	0	60	44	22	Microscopy	Blood and stool culture
Omoya et al. (2017)^96^	Nigeria	2015	Cross-sectional study	170 Pregnant women	16–45 years	170	79	79	0	112	33	35	Microscopy	Widal test
Onyido et al. (2014)^97^	Nigeria	2012	Cross-sectional study	200 Healthy individuals	1–80 years	52:148	10	10	0	50	40	11	Microscopy	Widal test
Orok et al. (2016)^98^	Nigeria	2015	Cross-sectional study	250 Febrile patients	1–75 years	113:137	2	2	0	202	200	NS	Microscopy	Blood culture
Oshiokhayamhe et al. (2021)^99^	Nigeria	NS	Cross-sectional study	200 Students	18–30 years	100:100	5	5	0	10	5	25	Microscopy	Widal test
Oundo et al. (2002)^60^	Kenya	1997–2001	Cross-sectional study	9147 Children with severe malaria	Mean 22.28 months (25.3)		101		101	9248	9147	Non-typhoidal *Salmonella* (352)	Microscopy	Blood culture
Ozumba et al. (2020)^100^	Nigeria	2015	Cross-sectional study	200 Pregnant women	< 20 to 60 years	200	8	8	0	16	8	78	Microscopy	Widal test
Pam et al. (2015)^101^	Nigeria	2015	Cross-sectional study	250 Pregnant women	< 20 to 60 years	250	9	9	0	16	7	68	RDT	Widal test
Pam et al. (2018)^61^	Nigeria	2015	Cross-sectional study	200 Pregnant women	< 20 (6), 21–30 (110), 31–40 (72), 41–50 (10), 51–60 (2)	All were females	9	9		25	16	86	Microscopy, RDT	Widal test
Park et al. (2016)^62^	Burkina Faso, Ethiopia, Ghana, Guinea-Bissau, Kenya, Madagascar, Senegal, South Africa, Sudan, and Tanzania	2010–2014	Cross-sectional study	497 Febrile patients	All age groups	NS	24	9	15		0	473	Microscopy, RDT	Blood culture
Phu et al. (2020)^63^	Vietnam	1991–2003	Cross-sectional study	845 Adult patients admitted with severe falciparum malaria	> 14 years	NS	4	3	1	840	836, mean age 31 years, range 15–79 years, median parasite count 81,766/μL (12,811 to 316,512/μL)	0	Microscopy	Blood culture
Popoola et al. (2019)^64^	Nigeria	2017	Cross-sectional study	682 Febrile patients	< 1 (7), 1–5 (217), 6–12 (189), 13–17 (61), 18–59 (198), ≥ 60 (10)	332:350	7	6	1	171	164	*S. typhi* (21), non-typhoidal *Salmonella* (5)	Microscopy	Molecular method
Qureshi et al. (2019)^65^	Pakistan	2012–2013	Cross-sectional study	1889 Febrile patients	All age groups	Infected cases 164:147	11	11		128	117 age 1–12 (67), 13–60 (50)	183, age 1–12 (110), 13–60 (73)	Microscopy	Rapid diagnostic test
Raja et al. (2016)^66^	India	2013–2014	Cross-sectional study	100 Febrile patients	NS	NS	2	2		10	8	6	Microscopy, RDT	Blood culture
Ramya et al. (2017)^67^	India	2010–2012	Cross-sectional study	824 Malaria, typhoid, dengue cases	NS	NS	55	55		NS	NS		RDT	Widal test
Sajid et al. (2017)^102^	Pakistan	NS	Cross-sectional study	300 Febrile patients	1 to > 46 years	150:150	21	21		65	44	16	Microscopy	Widal test
Sale et al. (2020)^103^	Nigeria	NS	Cross-sectional study	200 Febrile patients	≤ 10 to > 30 years	96:104	45	45	0	111	66	33	Microscopy, RDT	Widal test
Samatha et al. (2015)^68^	India	2014–2015	Cross-sectional study	582 Febrile patients	NS	NS	4	4		306	302	Widal test 132, culture 7	Microscopy	Blood culture
Sandlund et al. (2012)^69^	Sweden	1995–2009	Cross-sectional study	755 Malaria patients	Mean 33.6 (15.2 years), range 1–79 years (13.68 years), range 18–77 years	01:01.0	4	4		755	751	0	Microscopy, RDT, molecular Method	Blood culture
Shaikh et al. (2018)^70^	Pakistan	2017–2018	Cross-sectional study	985 Febrile patients	Malaria positive: mean 38.63	Malaria positive: 209:181	52	52		442	390	250	NS	Widal test
Sharma et al. (2016)^104^	India	2014–2016	Cross-sectional study	3010 Febrile patients	Children and adults	2260:750	48	48	0	210	162	12	Microscopy, RDT	Blood culture
Singh et al. (2014)^71^	India	2013	Cross-sectional study	1141 Febrile patients	≥ 12 years	618:523	1	1		147	146	92	Microscopy, RDT	Blood culture
Snehanshu et al. (2014)^105^	India	2012–2013	Cross-sectional study	200 Febrile patients	0 to > 60 years	88:112	5	5	0	36	31	NS	Microscopy	Blood culture
Sur et al. (2006)^106^	India	2004	Prospective study	3371 Febrile patients	0 to > 70 years	NS	3	3	0	93	90	92	Microscopy	Blood culture
Tabu et al. (2012)^72^	Kenya	2006–2009	Cross-sectional study	3578 Febrile (60 non-typhoidal *Salmonella* investigated for malaria)	NS	NS	12		12	NS	NS	Non-typhoidal *Salmonella* (48)	Microscopy	Blood culture
Tchuandom et al. (2018)^73^	Cameroon	2016–2017	Cross-sectional study	961 Febrile patients	≤ 15 years, mean 7.1 (2.9 years)	495:466	6	6		396	390	22	RDT	RDT
Ukaegbu et al. (2014)^107^	Nigeria	NS	Cross-sectional study	300 Febrile patients	0 to > 60 years	117:183	9	9	0	162	153	NS	Microscopy	Stool culture
Vats et al. (2018)^108^	India	NS	Cross-sectional study	300 Febrile patients	1–60 years	206:94	3	3	0	31	28	9	Microscopy	Blood culture
Verma et al. (2014)^74^	India	2012	Cross-sectional study	800 Febrile patients	NS	NS	9	9		NS	NS	NS	Microscopy, RDT	Blood culture
Walsh et al. (2000)^27^	Malawi	1996–1997	Cross-sectional study	128 Non-typhoidal *Salmonella*	0–13 years	NS	32	0	32	32	NS	96	Microscopy	Blood culture
Were et al. (2011)^28^	Kenya	2004–2006	Cross-sectional study	585 Children with *P. falciparum*	1–36 months	295:290	24	0	24	585	561	NS	Microscopy	Blood culture

*ELISA* enzyme-linked immunosorbent assay, *NS* not specified, *RDT* rapid diagnostic test.

Among the 81 studies included in the analysis, 49 studies (60.5%) enrolled febrile patients^35–41,43–47,50–53,55,57,59,62,64–68,70–77,81,83,84,87,89,90,92,93,95,98,102–108^, 8 studies enrolled pregnant women^42,61,82,91,94,96,100,101^, 6 studies enrolled patients with severe malaria^30,54,63,79,80,85^, 9 studies enrolled malaria positive patients^28,48,56,58,60,69,78,86,88^, 5 studies enrolled typhoid/non-typhoid-positive patients^17,27,29,31,49^, and one study enrolled children with pathogenic bacteremia^20^. Co-infections with malaria and typhoidal/NTS were reported in 4,523 cases from 73,775 total patients enrolled in the 81 included studies. Co-infections with malaria and typhoidal *Salmonella* spp., including *S. typhi* and *S. paratyphi*, were reported in 3813 cases from 56 studies^31,36,38,39,41–43,45–48,50–52,57,59,61,65–68,70,71,73–78,81–84,86–108^. Co-infections with malaria and NTS, such as *S. typhimurium*, were reported in 707 cases from 18 studies^17,27–31,44,53,56,60,62,69,72,79,80,84,85,93^. Co-infections with malaria and both typhoidal and NTS spp. were reported in 13 studies^20,31,35,37,40,49,54,55,58,63,64,84,93^.

*Salmonella* spp. infection was identified using blood cultures (39/81, 48.1%)^17,20,27–31,37,38,44,47,49,50,53,54,56–58,60,62,63,66,68,69,71,72,74,77–81,83,85,89,98,104–106,108^, Widal test (27/81, 33.3%)^36,40,41,43,45,46,48,51,52,59,61,67,70,75,86–88,90,94,96,97,99–103^, stool cultures (5/81, 6.17%)^82,84,92,93,107^, RDTs (4/81, 4.94%)^35,42,73,91^, and molecular methods (3/43, 6.98%)^39,55,64^. One study^95^ employed both blood and stool cultures. Some studies used combinations of methods to identify *Salmonella* spp. infection. However, only a definitive method was demonstrated in this qualitative synthesis. For the identification of malaria, *Plasmodium* spp. infections were identified via microscopy alone (52/81, 64.2%)^17,27,28,30,31,36–38,40,43,45–47,50–53,56,59,60,63–65,68,72,75,77,79–87,89,90,92–100,102,105–108^, microscopy/RDT (16/81, 19.8%)^20,41,44,49,54,58,61,66,69,71,74,78,88,91,103,104^, RDT alone (5/81, 6.17%)^57,67,73,76,101^, molecular method^39,55^, microscopy/RDT/molecular method^35,69^, not specified^42,48,70^, and ELISA^29^.

Among the 81 studies included in the present study, 30 (37%) were rated as low risk of bias, whereas 51 studies had a moderate risk of bias (51/81, 63%). Studies with a high risk of bias were removed during the study selection (Supplementary Table S2).

### Prevalence of typhoidal and NTS and malaria co-infections among febrile patients

**Figure 2:**
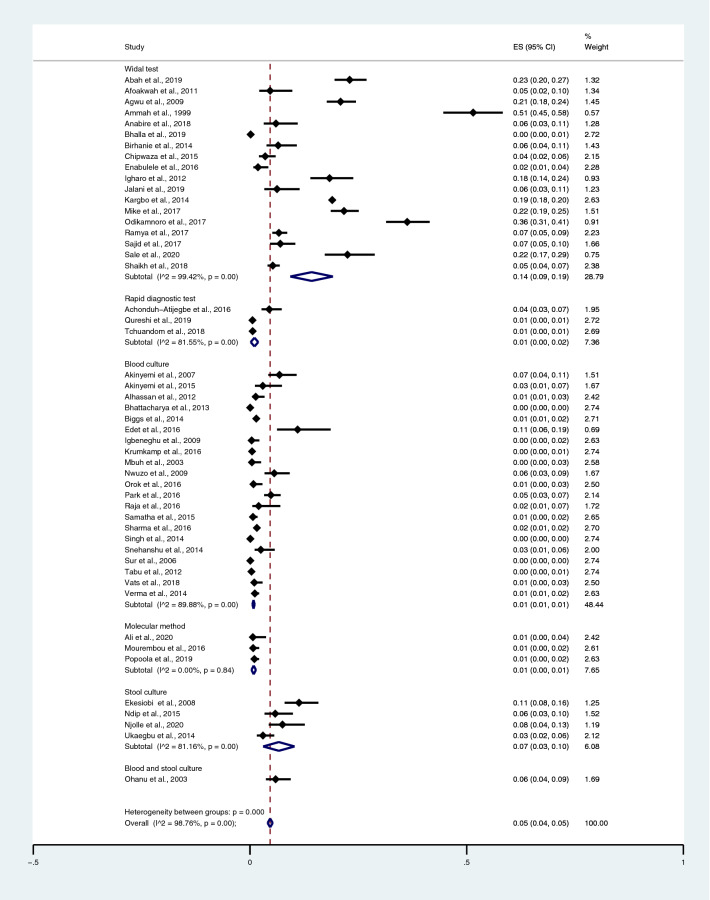
The pooled prevalence of typhoidal/NTS and malaria co-infection among febrile patients detected using diagnostic tests for *Salmonella* spp. *ES* proportion estimate (multiply 100 units for interpreted as prevalence estimate), *CI* confidence interval.

The pooled prevalence rate of typhoidal/NTS and malaria co-infections among febrile patients was estimated from 50 studies^35–41,43–47,50–53,55,57,59,62,64–68,70–77,81,83,84,87,89,90,92,93,95,98,102–108^. The studies were divided into four groups based on diagnostic tests for *Salmonella* spp. The results indicated that the pooled prevalence rates of typhoidal/NTS and malaria co-infections among febrile patients were 14% (95% CI 9–19%; I^2^, 99.4%) using the Widal test, 1% (95% CI 1–1%; I^2^, 89.9%) using blood culture, 1% (95% CI 0–2%; I^2^, 81.6%) using RDTs, 1% (95% CI 0–1%; I^2^, 0%) using a molecular method, 7% (95% CI 3–10%; I^2^, 81.2%) using stool cultures, and 6% (95% CI 4–9%) using a combination of blood and stool cultures (Fig. 2).

**Figure 3:**
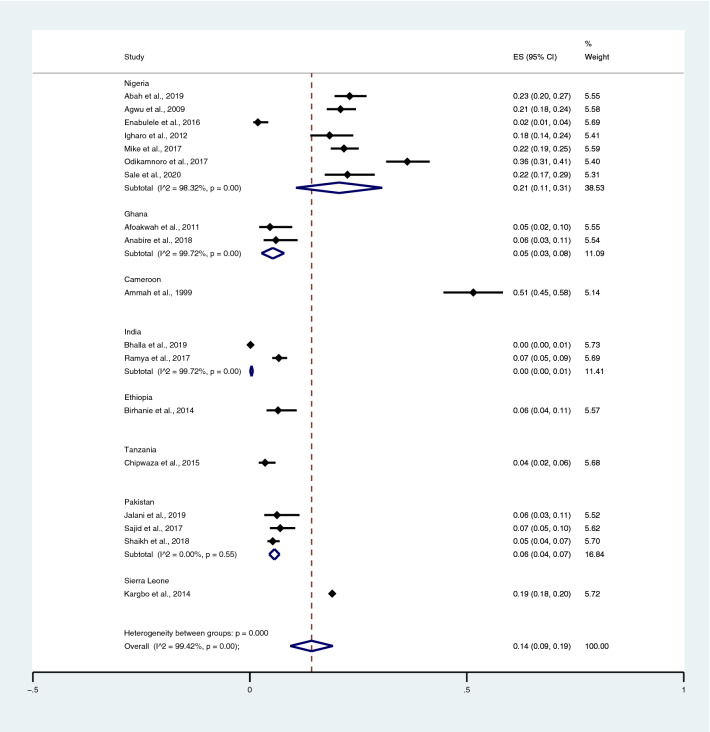
Pooled prevalence of typhoidal/NTS and malaria co-infection using the Widal test for the identification of *Salmonella* spp. infection stratified by countries. *ES* proportion estimate (multiply 100 units for interpreted as prevalence estimate), *CI* confidence interval.

When *Salmonella* spp. infections were detected using the Widal test, the highest prevalence rate of co-infections was noted in Cameroon (51%; 95% CI 45–58%) and Nigeria (21%; 95% CI 11–31%; I^2^, 98.3%), whereas lower prevalence rates were detected in Sierra Leone (19%; 95% CI 18–20%), Ethiopia (6%; 95% CI 4–11%), Pakistan (6%; 95% CI 4–7%; I^2^, 0%), Ghana (5%; 95% CI 3–8%; I^2^, 99.7%), India (0%; 95% CI 0–1%; I^2^, 99.7%), and Tanzania (4%; 95% CI 2–6%) (Fig. 3).

**Figure 4:**
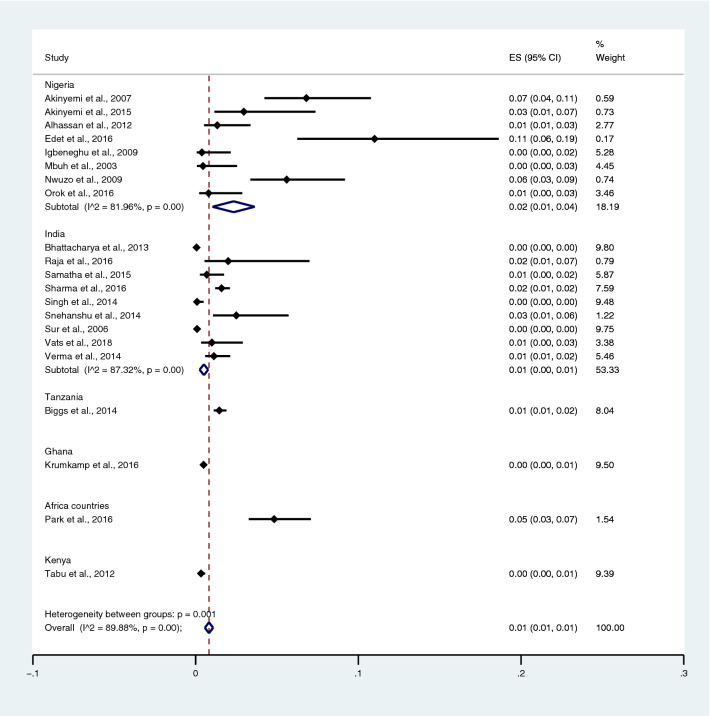
Pooled prevalence of typhoidal/NTS and malaria co-infection using blood cultures for the identification of *Salmonella* spp. infection stratified by countries. *ES* proportion estimate (multiply 100 units for interpreted as prevalence estimate), *CI* confidence interval, *NS* not specified.

Among the studies using blood culture for the identification of *Salmonella* spp. infections, the highest prevalence of co-infection was reported in Burkina Faso, Ethiopia, Ghana, Guinea-Bissau, Kenya, Madagascar, Senegal, South Africa, Sudan, and Tanzania (5%; 95% CI 3–7%). Contrarily, lower prevalence was reported in Nigeria (2%; 95% CI 1–4%; I^2^, 82%), India (1%; 95% CI 0–1%; I^2^, 87.3%), Tanzania (1%; 95% CI 1–2%), Ghana (0%; 95% CI 0–1%), and Kenya (0%; 95% CI 0–1%) (Fig. 4).

**Figure 5:**
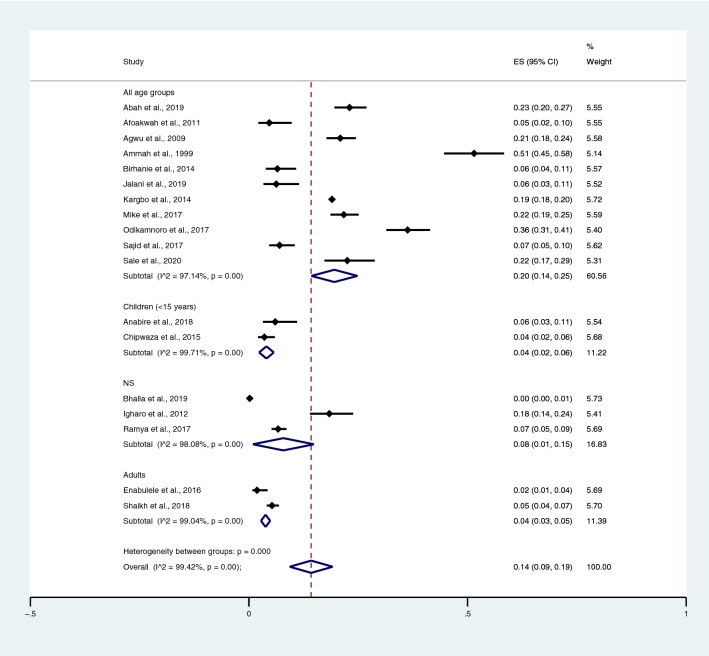
Pooled prevalence of typhoidal/NTS and malaria co-infection using the Widal test for the identification of *Salmonella* spp. infection stratified by age groups. *ES* proportion estimate (multiply 100 units for interpreted as prevalence estimate), *CI* confidence interval.

**Figure 6:**
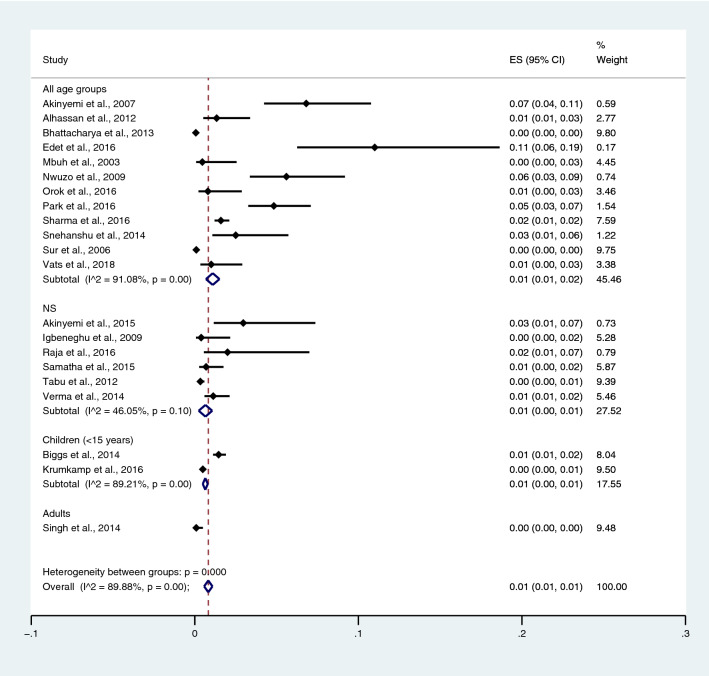
Pooled prevalence of typhoidal/NTS and malaria co-infection using blood cultures for the identification of *Salmonella* spp. infection stratified by age groups. *ES* proportion estimate (multiply 100 units for interpreted as prevalence estimate), *CI* confidence interval, *NS* not specified.

Among the studies using the Widal test for the identification of *Salmonella* spp. infections, the highest prevalence of co-infections was noted in the studies that enrolled participants in all age groups (95% CI 20%; 95% CI 14–25%; I^2^, 97.1%). The prevalence of co-infections was 4% in children (95% CI 2–6%; I^2^, 99.7%), 8% in the not specified (NS) age group (95% CI 1–15%), and 4% in adults (95% CI 3–5%; I^2^, 99%) (Fig. 5) when *Salmonella* spp. infections were detected using the Widal test. Among the studies using blood cultures for the identification of *Salmonella* spp. infections, the prevalence of co-infections was 1% in all age groups (95% CI 1–2%; I^2^, 91.1%), 1% in the NS group (95% CI 0–1%; I^2^, 46.1%), and 1% in children (95% CI 0–1%; I^2^, 89.2%) (Fig. 6).

**Figure 7:**
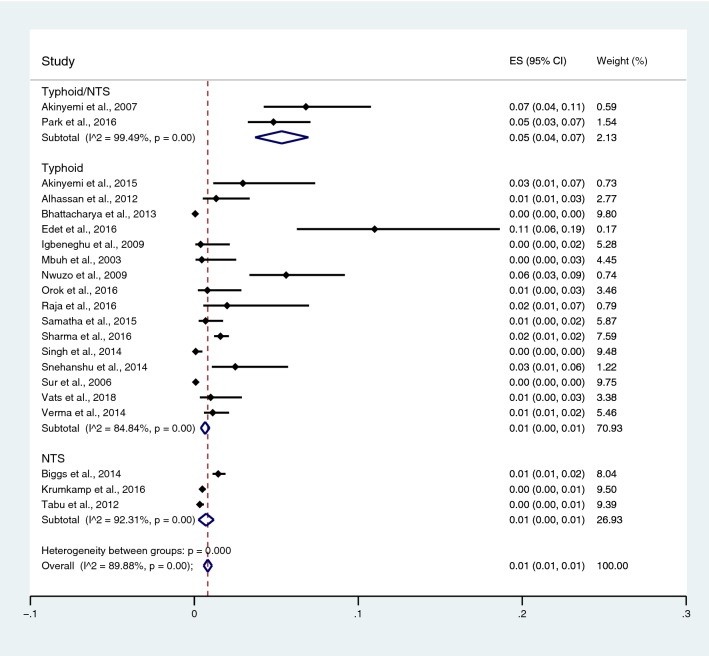
Pooled prevalence of typhoidal/NTS and malaria co-infection using blood cultures for the identification of *Salmonella* spp. infection stratified by typhoidal/NTS infection. *ES* proportion estimate (multiply 100 units for interpreted as prevalence estimate), *CI* confidence interval, *NS* not specified.

**Figure 8:**
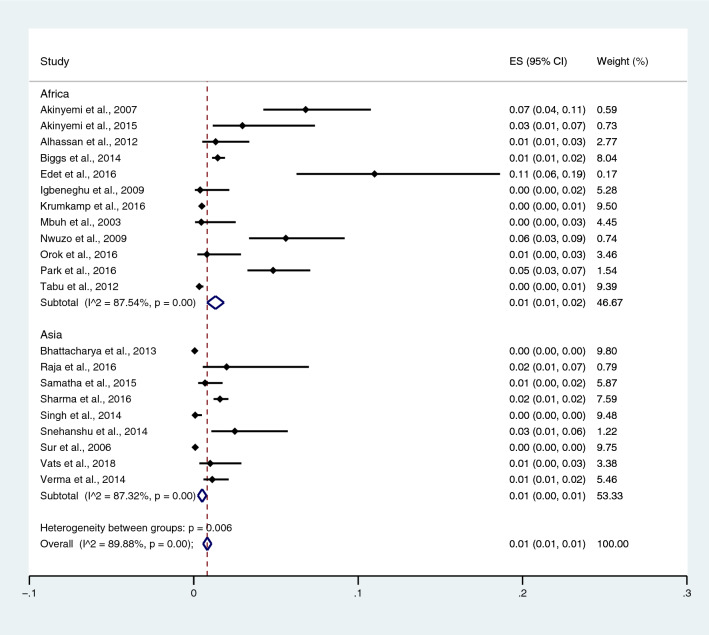
Pooled prevalence of typhoidal/NTS and malaria co-infection using blood cultures for the identification of *Salmonella* spp. infection stratified by regions. *ES* proportion estimate (multiply 100 units for interpreted as prevalence estimate), *CI* confidence interval, *NS* not specified.

**Figure 9:**
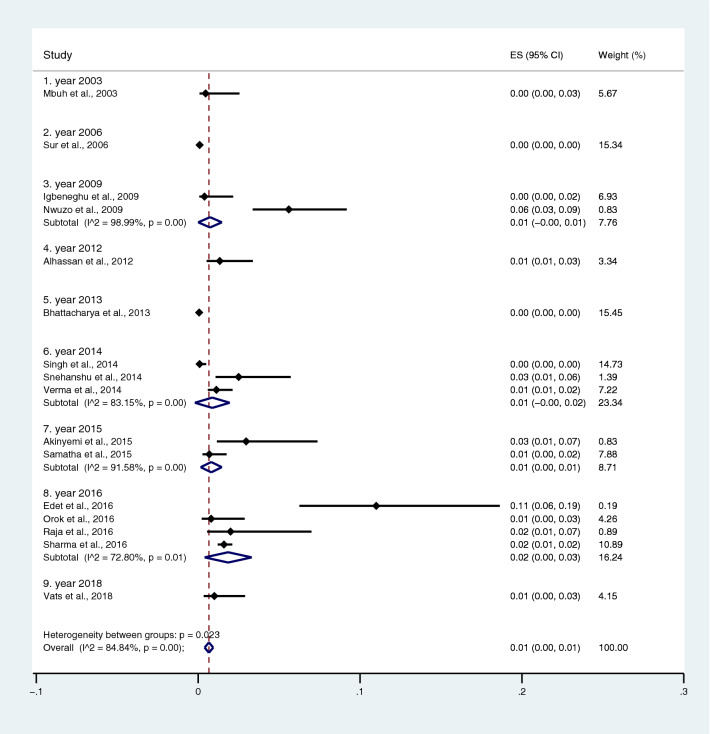
Pooled prevalence of typhoidal and malaria co-infection using blood cultures for the identification of *Salmonella* spp. infection stratified by time (publication year). *ES* proportion estimate (multiply 100 units for interpreted as prevalence estimate), *CI* confidence interval, *NS* not specified.

**Figure 10:**
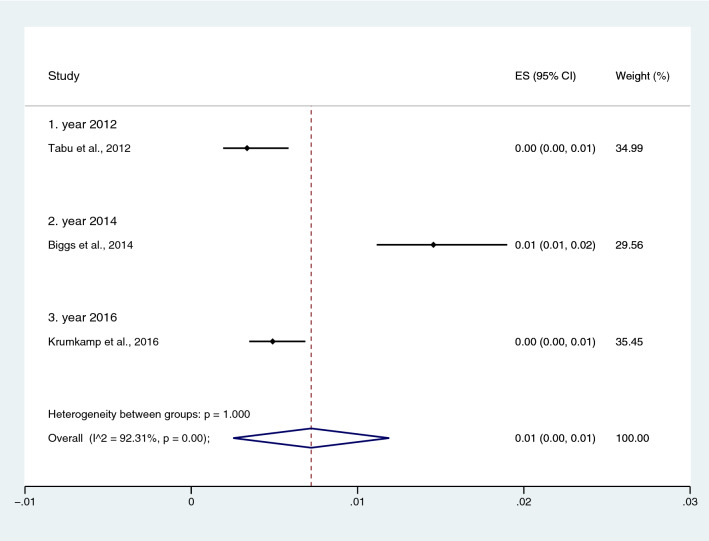
Pooled prevalence of NTS and malaria co-infection using blood cultures for the identification of *Salmonella* spp. infection stratified by time (publication year). *ES* proportion estimate (multiply 100 units for interpreted as prevalence estimate), *CI* confidence interval, *NS* not specified.

Subgroup analysis of typhoidal/NTS infection, regions (Africa and Asia), and time (publication year) was performed using the data from studies using blood culture for typhoidal/NTS identification^37,38,44,50,53,57,62,66,68,71,72,74,77,81,83,89,98,104–106,108^. Results showed that the prevalence rates of malaria and typhoid co-infections and malaria and NTS co-infections were 1% (95% CI 0–1%; I^2^, 84.8%) and 1% (95% CI 0–1%; I^2^, 92.3%) (Fig. 7). Subgroup analysis of regions showed that the prevalence rates of malaria and typhoidal/NTS co-infections were 1% in Africa (95% CI 1–2%; I^2^, 87.5%) and 1% in Asia (95% CI 0–1%; I^2^, 87.3%) (Fig. 8). Subgroup analysis of time showed that the prevalence rate of malaria and typhoid co-infections was highest (2%) in 2016 (95% CI 0–1%; I^2^, 72.8%) and 1% in 2009, 2012, 2014, and 2015 and 0% in 2003, 2006, 2013, and 2018 (Fig. 9). Subgroup analysis of time showed that the prevalence rate of malaria and NTS co-infections was highest (1%) in 2014 and 0% in 2012 and 2016 (Fig. 10).

### Prevalence of typhoidal/NTS infections among patients with malaria

**Figure 11:**
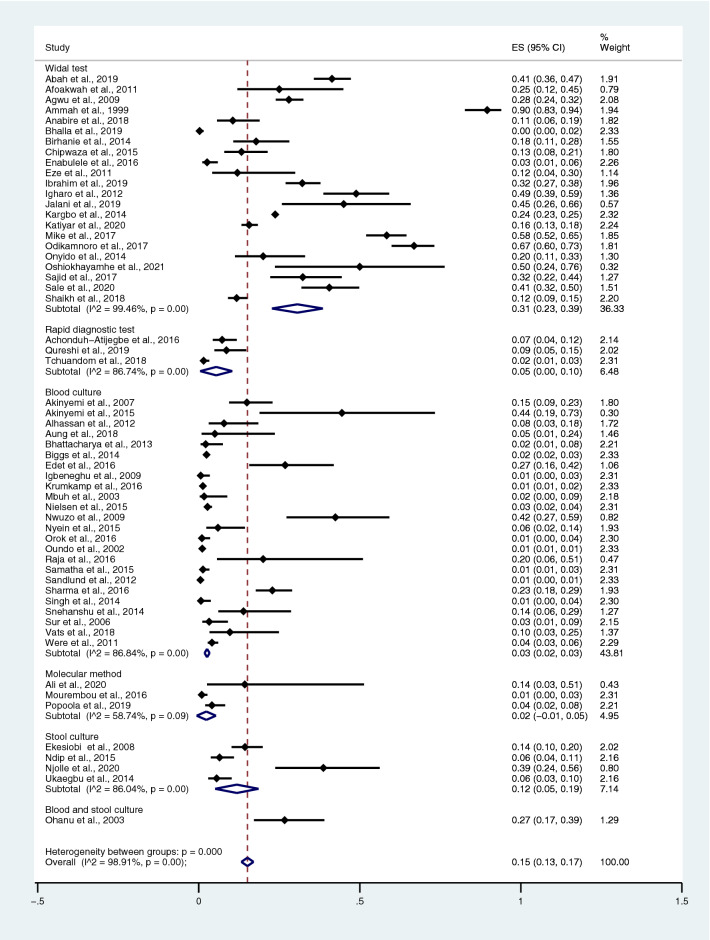
Prevalence of typhoidal/NTS infection among patients with malaria detected using diagnostic tests for *Salmonella* spp. *ES* proportion estimate (multiply 100 units for interpreted as prevalence estimate), *CI* confidence interval.

The pooled prevalence rate of typhoidal/NTS infection among malaria patients was estimated from 57 studies^28,35–41,43–48,50–53,55–60,64–66,68–71,73,75–78,81,83,84,86–90,92,93,95,97–99,102–108^. The studies were divided into groups based on diagnostic tests for *Salmonella* spp. The pooled prevalence rates of typhoidal/NTS infection among patients with malaria were 31% (95% CI 23–39%; I^2^, 99.5%) using the Widal test, 5% (95% CI 0–10%; I^2^, 86.7%) using RDTs, 3% (95% CI 2–3%; I^2^, 86.8%) using blood culture, 2% (95% CI − 1 to 5%; I^2^, 58.7%) using molecular methods, 12% (95% CI 5–19%; I^2^, 86%) using stool cultures, and 27% (95% CI 17–39%) using a combination of blood and stool cultures (Fig. 11).

**Figure 12:**
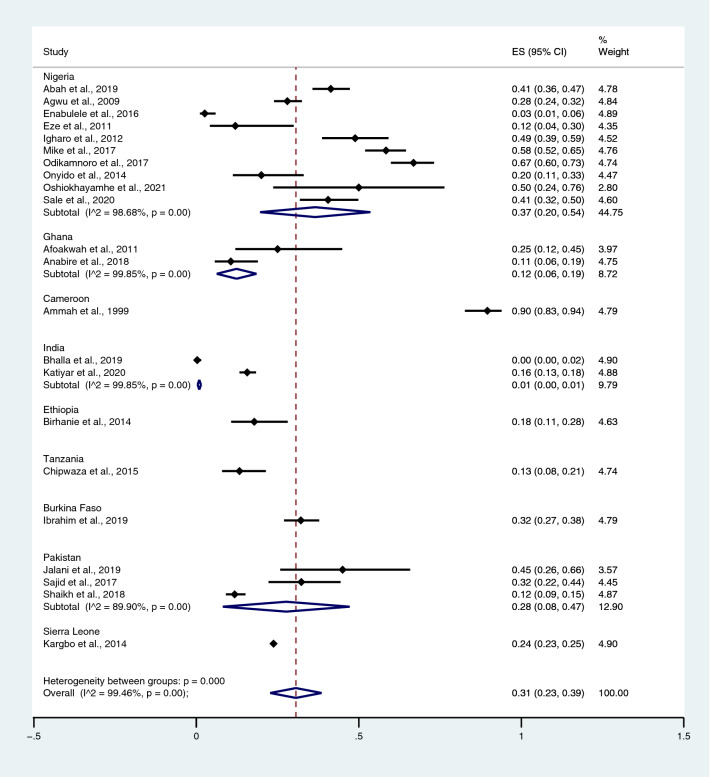
Prevalence of typhoidal/NTS infection among patients with malaria using the Widal test for the identification of *Salmonella* spp. infection stratified by countries. *ES* proportion estimate (multiply 100 units for interpreted as prevalence estimate), *CI* confidence interval.

**Figure 13:**
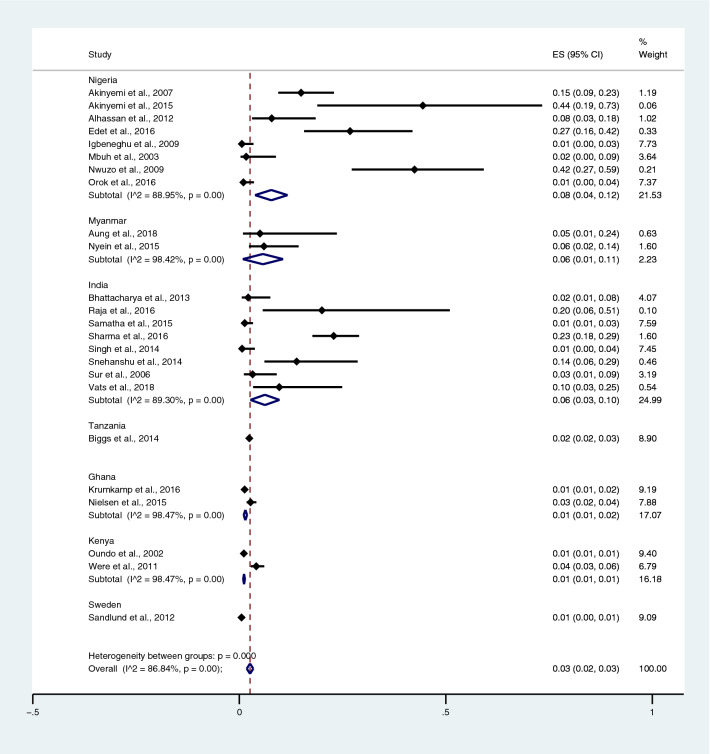
Prevalence of typhoidal/NTS infection among patients with malaria detected using blood cultures for the identification of *Salmonella* spp. infection stratified by countries. *ES* proportion estimate (multiply 100 units for interpreted as prevalence estimate), *CI* confidence interval.

Among the studies using the Widal test for the identification of *Salmonella* spp. infections, the highest prevalence rate of typhoidal/NTS among patients with malaria was reported in Cameroon (90%; 95% CI 83–94%) and Nigeria (37%; 95% CI 20–54%; I^2^, 98.7%), whereas lower prevalence rates were reported in Burkina Faso (32%; 95% CI 27–38%), Pakistan (28%; 95% CI 8–47%; I^2^, 89.9%), Sierra Leone (24%; 95% CI 23–25%), Ethiopia (18%; 95% CI 11–28%), Tanzania (13%; 95% CI 8–21%), Ghana (12%; 95% CI 6–19%; I^2^, 99.9%), and India (1%; 95% CI 0–1%) (Fig. 12). Among the studies using hemoculture for the identification of *Salmonella* spp. infections, the highest prevalence rate of typhoidal/NTS among patients with malaria was reported in Nigeria (8%; 95% CI 4–12%; I^2^, 89%), whereas lower prevalence rates were reported in Myanmar (6%; 95% CI 1–11%; I^2^, 98.4%), India (6%; 95% CI 3–10%; I^2^, 89.3%), Tanzania (2%; 95% CI 2–3%), Ghana (1%; 95% CI 1–2%; I^2^, 97.9%), Kenya (1%; 95% CI 1–1%; I^2^, 98.5%), and Sweden (1%; 95% CI 0–1%) (Fig. 13).

**Figure 14:**
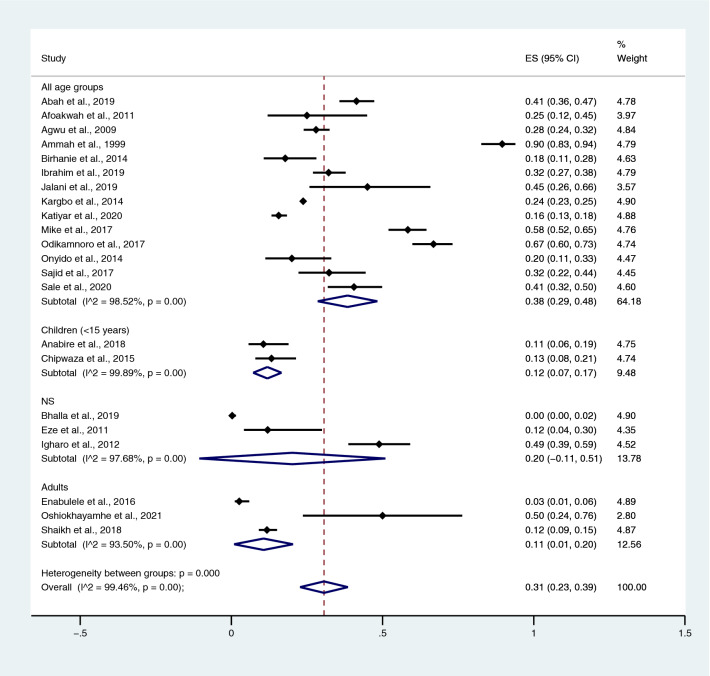
Prevalence of typhoidal/NTS infection among patients with malaria using the Widal test for the identification of *Salmonella* spp. infection stratified by age groups. *ES* proportion estimate (multiply 100 units for interpreted as prevalence estimate), *CI* confidence interval, *NS* not specified.

**Figure 15:**
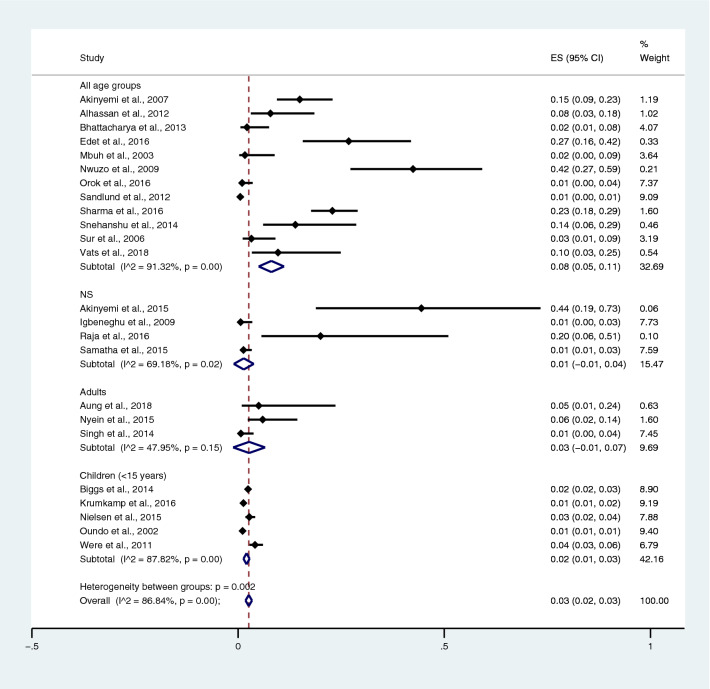
Prevalence of typhoidal/NTS infection among patients with malaria using blood cultures for the identification of *Salmonella* spp. infection stratified by age groups. *ES* proportion estimate (multiply 100 units for interpreted as prevalence estimate), *CI* confidence interval, *NS* not specified.

**Figure 16:**
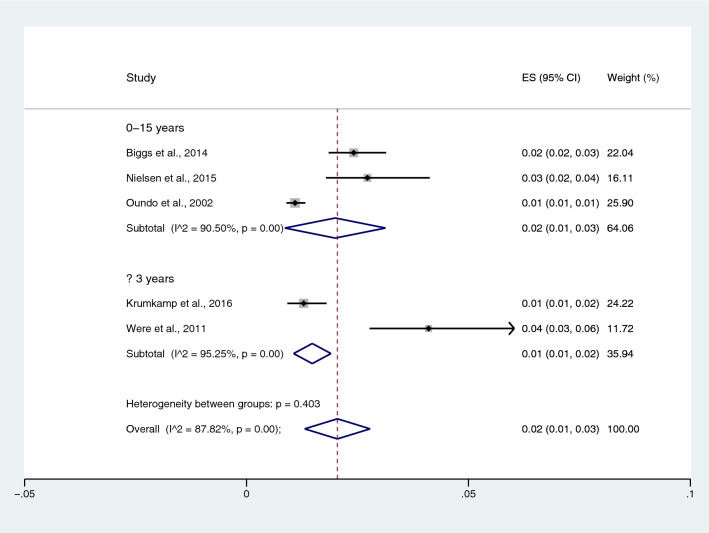
Prevalence of NTS infection among patients with malaria using blood cultures for the identification of *Salmonella* spp. infection stratified by age groups. *ES* proportion estimate (multiply 100 units for interpreted as prevalence estimate), *CI* confidence interval, *NS* not specified.

Among the studies using the Widal test for the identification of *Salmonella* spp. infections, the prevalence rates of typhoidal/NTS among patients with malaria in all age groups were 38% (95% CI 29–48%; I^2^, 98.5%), 12% in children (95% CI 7–17%; I^2^, 99.9%), 20% in the NS age group (95% CI − 11 to 51%; I^2^, 97.7%), and 11% in adults (95% CI 1–20%; I^2^, 93.5%) (Fig. 14). Among the studies using blood culture for the identification of *Salmonella* spp. infections, the prevalence rates of typhoidal/NTS among malarial patients were 8% in all age groups (95% CI 5–11%; I^2^, 91.3%), 1% in the NS age group (95% CI − 1 to − 4%; I^2^, 69.2%), 3% in adults (95% CI − 1 to 7%; I^2^, 48%), and 2% in children (95% CI 1–3%; I^2^, 87.8%) (Fig. 15). Subgroup analysis of age (≤ 3 years and 0–15 years) of NTS infection among patients with malaria was performed using the data of five studies^28,44,53,56,60^. Results showed that the prevalence rates of NTS infection among patients with malaria were 2% in patients aged 0–15 years (95% CI − 1 to − 43%; I^2^, 90.5%) and 1% in patients aged ≤ 3 years (95% CI 1–2%; I^2^, 95.3%) (Fig. 16).

**Figure 17:**
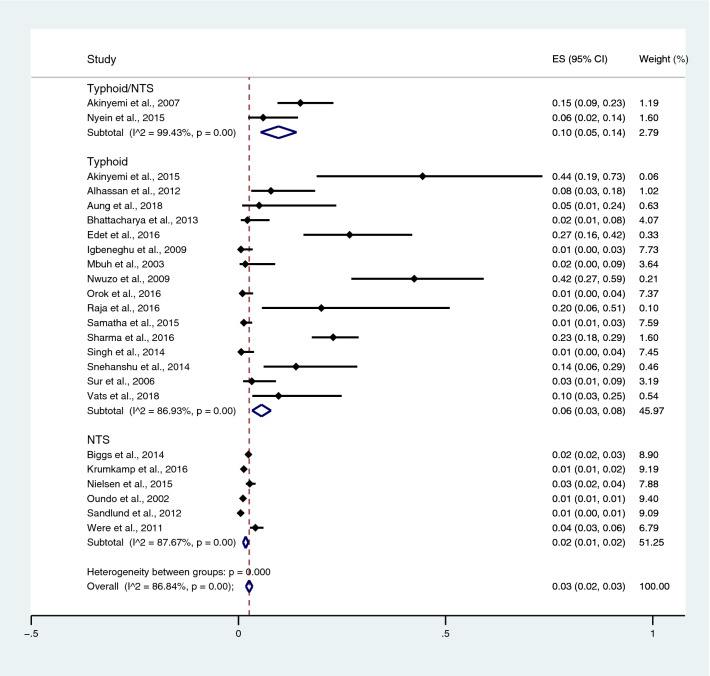
Prevalence of typhoidal/NTS infection among patients with malaria using blood cultures for the identification of *Salmonella* spp. infection stratified by typhoidal/NTS infection. *ES* proportion estimate (multiply 100 units for interpreted as prevalence estimate), *CI* confidence interval, *NS* not specified.

**Figure 18:**
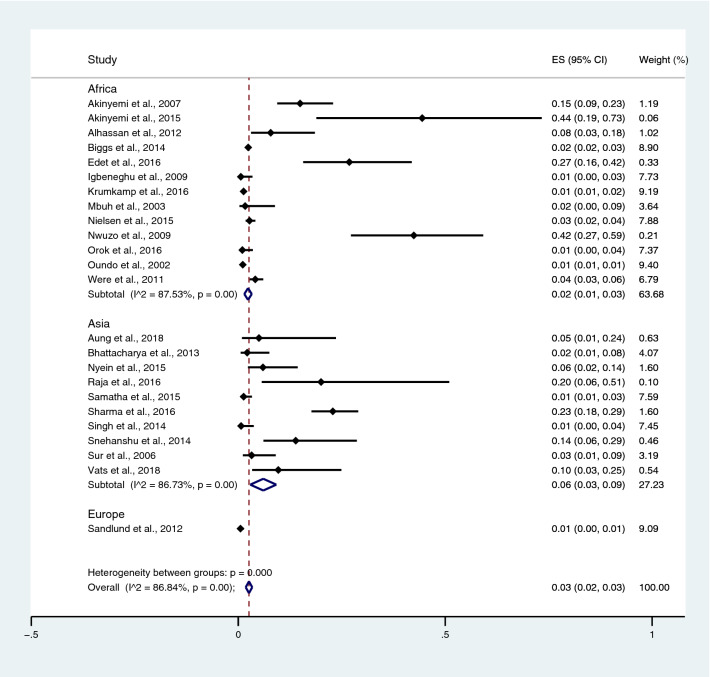
Prevalence of typhoidal/NTS infection among patients with malaria using blood cultures for the identification of *Salmonella* spp. infection stratified by regions. *ES* proportion estimate (multiply 100 units for interpreted as prevalence estimate), *CI* confidence interval, *NS* not specified.

**Figure 19:**
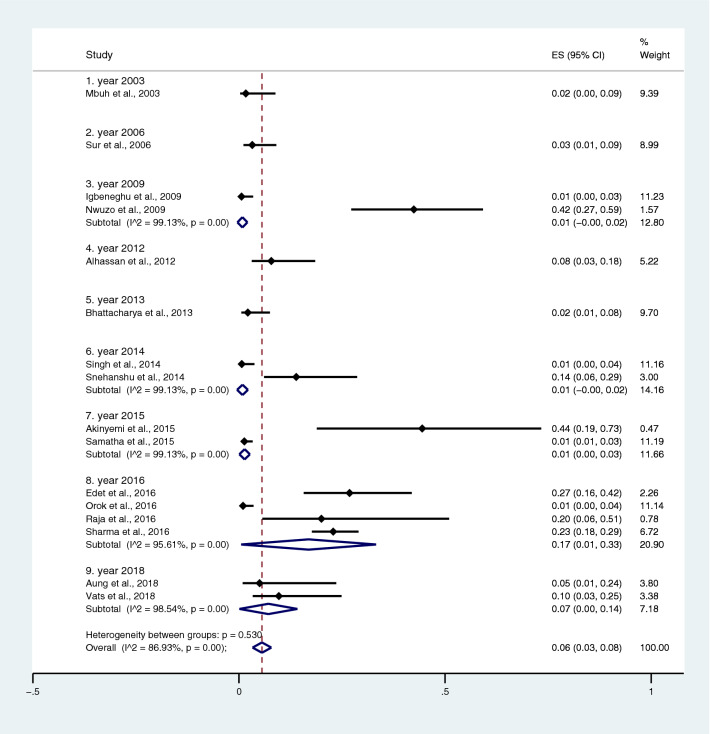
Prevalence of *Salmonella* spp. infection among patients with malaria using blood cultures for the identification of *Salmonella* spp. infection stratified by time (publication years). *ES* proportion estimate (multiply 100 units for interpreted as prevalence estimate), *CI* confidence interval, *NS* not specified.

**Figure 20:**
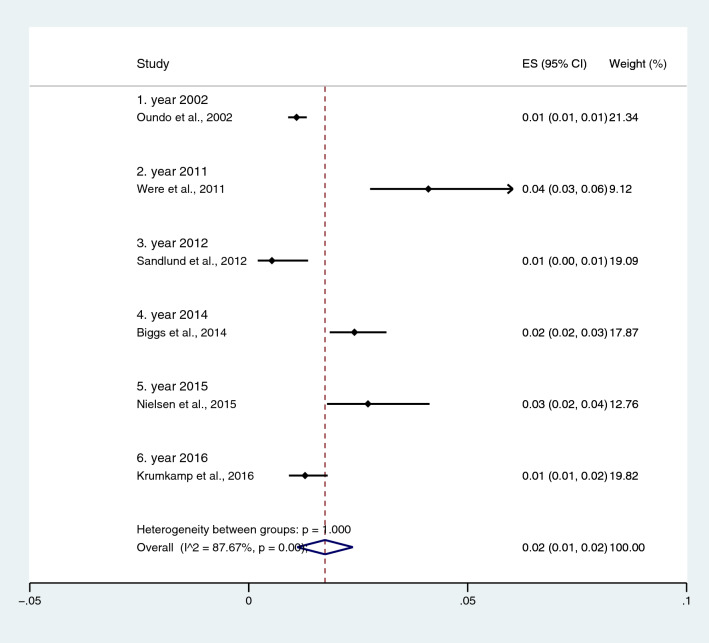
Prevalence of NTS infection among patients with malaria using blood cultures for the identification of *Salmonella* spp. infection stratified by time (publication years). *ES* proportion estimate (multiply 100 units for interpreted as prevalence estimate), *CI* confidence interval, *NS* not specified.

Subgroup analysis of typhoidal/NTS, regions (Africa and Asia), and time (publication year) was performed using the data from studies using blood culture for typhoidal/NTS identification^28,37,38,44,50,53,56–58,60,66,68,69,71,77,78,81,83,89,98,104–106,108^. Results showed that the prevalence rates of typhoidal and NTS infection among patients with malaria were 6% (95% CI 3–8%; I^2^, 86.9%) and 2% (95% CI 1–2%; I^2^, 87.7%) (Fig. 17). Subgroup analysis of regions showed that the prevalence rates of typhoidal/NTS among patients with malaria were 2% in Afica (95% CI 1–3%; I^2^, 87.5%), 6% in Asia (95% CI 3–9%; I^2^, 86.7%), and 1% in Europe (95% CI 0–1%) (Fig. 18). Subgroup analysis of time showed that the prevalence rate of typhoidal *Salmonella* infection among patients with malaria was highest (17%) in 2016 (95% CI 1–33%; I^2^, 95.6%), 8% in 2012 (95% CI 3–18%) and 7% in 2018 (95% CI 0–14%; I^2^, 98.5%). The low prevalence of typhoidal *Salmonella* infection infection among patients with malaria was demonstrated in 2016 (13%), 2003 (2%), 2013 (2%), and 2009 (1%) (Fig. 19). Subgroup analysis of time showed that the prevalence rates of NTS infection among patients with malaria were highest in 2011 (4%), 2015 (3%), and 2014 (2%) and low in 2014 (2%), 2002 (1%), 2012 (1%), and 2016 (1%) (Fig. 20).

### Comparison of typhoidal/NTS infections among patients with severe and non-severe malaria

**Figure 21:**
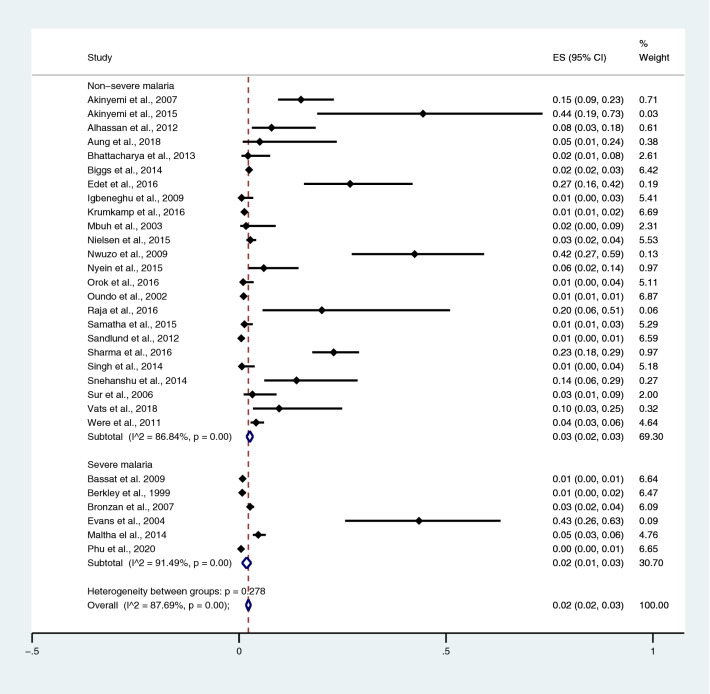
Pooled prevalence of typhoidal/NTS infection among patients with severe and non-severe malaria. *ES* proportion estimate (multiply 100 units for interpreted as prevalence estimate), *CI* confidence interval.

The pooled prevalence rates of typhoidal/NTS infections among patients with severe and non-severe malaria were estimated using data from 24 studies that enrolled patients with non-severe malaria^28,37,38,44,50,53,56–58,60,66,68,69,71,77,78,81,83,89,98,104–106,108^ and 6 studies that enrolled patients with severe malaria^30,54,63,79,80,85^. All 30 studies employed the blood culture method to identify *Salmonella* spp. infections. The pooled prevalence rates of typhoidal/NTS infection were 2% in patients with severe malaria (95% CI 1–3%; I^2^, 91.5%) and 3% in patients with non-severe malaria (95% CI 2–3%; I^2^, 86.8%) (Fig. 21).

### Prevalence of malaria infections among patients with typhoidal/NTS infections

**Figure 22:**
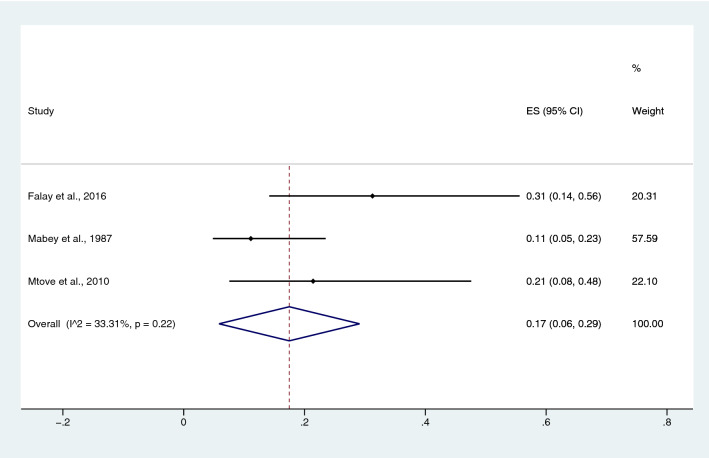
Pooled prevalence of malaria infection among patients with typhoidal *Salmonella* spp. *ES* proportion estimate (multiply 100 units for interpreted as prevalence estimate), *CI* confidence interval.

**Figure 23:**
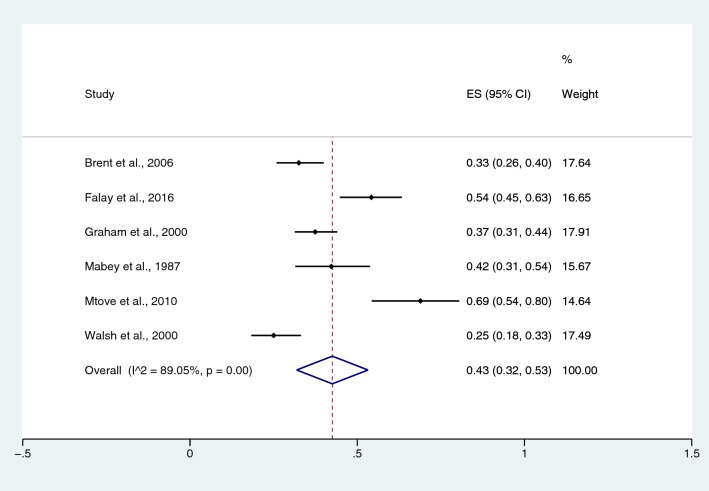
Pooled prevalence of malaria infection among patients with NTS. *ES* proportion estimate (multiply 100 units for interpreted as prevalence estimate), *CI* confidence interval.

The pooled prevalence rate of malaria infections among patients with typhoidal *Salmonella* spp. infection was estimated from three studies^20,31,49^. The pooled prevalence rate of malaria infection in patients with typhoidal *Salmonella* spp. was 17% in children (95% CI 6–29%; I^2^, 33.3%) (Fig. 22). The pooled prevalence rate of malaria infection among patients with NTS, which was estimated from six studies^17,20,27,29,31,49^, was 43% in children (95% CI 32–53%; I^2^, 89.1%) (Fig. 23).

### Probability of *Plasmodium* spp. and *Salmonella* spp. co-infections

**Figure 24:**
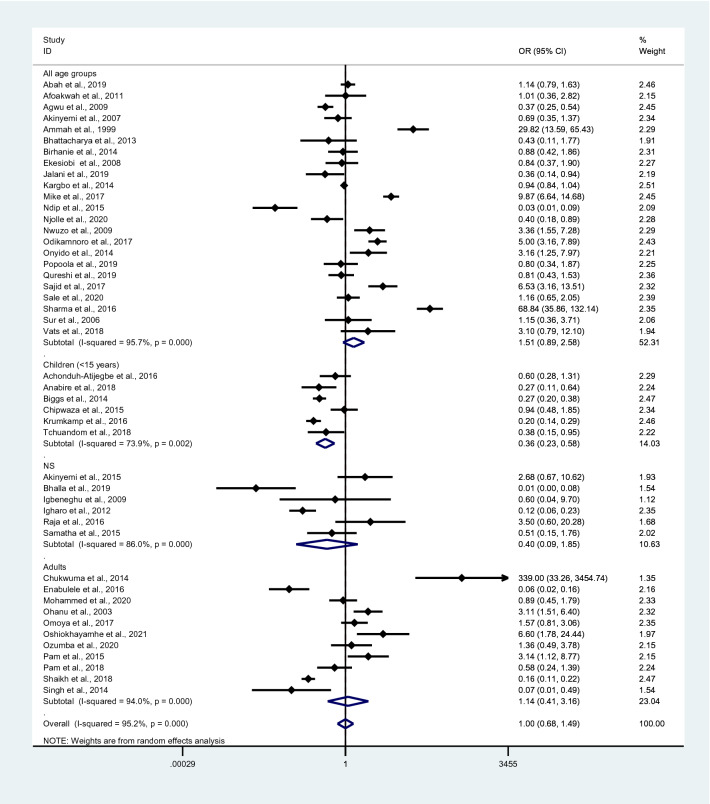
Probability of *Plasmodium* spp. and *Salmonella* spp. co-infections. *OR* odds ratio, *CI* confidence interval, *NS* not specified.

The probability of *Plasmodium* spp. and *Salmonella* spp. co-infections was estimated from 46 studies^35–38,40,41,43–47,50–53,57,59,61,64–66,68,70,71,73,75,76,81,82,84,87,90–93,95–97,99–104,106,108^, which reported the following parameters: total number of *Plasmodium* spp. and *Salmonella* spp. co-infections, total number of malaria, total number of malaria without typhoid, and total number of febrile patients without malaria/typhoid. *Plasmodium* spp. and *Salmonella* spp. co-infections in all age groups occurred by chance (p = 0.126; odds ratio, 1.51; 95% CI 0.89–2.58; I^2^, 95.7%), whereas *Plasmodium* spp. and *Salmonella* spp. co-infections in children did not (p < 0.0001; odds ratio, 0.36; 95% CI 0.23–0.58; I^2^, 73.9%). No association between *Plasmodium* spp. and *Salmonella* spp. infections was observed in the NS age group (p, 0.24; odds ratio, 0.40; 95% CI 0.09–1.85; I^2^, 86%) or adults (p, 0.799; odds ratio, 1.14; 95% CI 0.41–3.16; I^2^, 94%). Overall, *Plasmodium spp*. and *Salmonella* spp. co-infection occurred by chance (p, 0.987; odds ratio, 1.00; 95% CI 0.68–1.49; I^2^, 95.2%) (Fig. 24). A significantly higher odds ratio of co-infection was reported in Nigeria^57,59,82,90,95,97,101^, Cameroon^40^, India^104^, and Pakistan^102^, whereas a significantly lower odds ratio of co-infection was found in Nigeria^36,47,51^, Cameroon^73,92,93^, Pakistan^52,70^, Ghana^41,53^, Tanzania^44^, Kenya^60^, and India^43,71^.

### Outcomes of malaria and typhoidal/NTS co-infections

A limited number of studies reported clinical outcomes of patients with co-infections (Table S2). Five studies^60,63,79,80,92^ reported outcomes of co-infection. Among those studies, three studies^60,80,92^ reported outcomes of malaria and NTS co-infections, and one study^63^ reported outcomes of malaria and typhoid co-infections. The case fatality rate in patients with malaria and NTS co-infections was 16% (95% CI 9–24%; I^2^, 89.1%; three studies), while one study^63^ reported the case fatality rate in patients with malaria and typhoidal *Salmonella* co-infections at 33% (95% CI 6–79%) (Supplementary Fig. S1). The difference in malarial parasitemia between co-infections and *Plasmodium* spp. monoinfection was estimated by two studies^79,80^. Results showed a higher mean of malarial parasitemia in patients with co-infections than those with *Plasmodium* spp. monoinfection (p, 0.023; WMD, 7926.7 parasites⁄µL of blood (95% CI 1091–14,762.3 parasites⁄µL of blood; I^2^, 0%, two studies) (Supplementary Fig. S2). The study by Bassat et al.^79^ showed a lower rate of respiratory distress in patients with co-infections (4/12, 33.3%) than those with *Plasmodium* spp. monoinfection (542/1328, 40.8%). The study by Bassat et al.^79^ also showed a lower mean hematocrit in patients with co-infections (22.1 ± 9.3%, 12 cases) than those with *Plasmodium* spp. monoinfection (23.4 ± 8.4%, 1328 cases).

### Sensitivity test

After excluding outliers^40,82,104^, the probability of *Plasmodium* spp. and *Salmonella* spp. co-infection was estimated from 43 studies^35–38,41,43–47,50–53,57,59,61,64–66,68,70,71,73,75,76,81,84,87,90–93,95–97,99–103,106,108^. Overall, *Plasmodium* spp. and *Salmonella* spp. co-infection occurred by chance (p, 0.148; odds ratio, 0.77; 95% CI 0.54–1.10; I^2^, 93.6%) (Supplementary Fig. S3). However, the use of a fixed-effects model in the meta-analysis indicated that *Plasmodium* spp. and *Salmonella* spp. co-infection did not occur by chance (p < 0.0001; odds ratio, 0.82; 95% CI 0.76–0.88; I^2^, 93.6%) (Supplementary Fig. S4).

### Publication bias

Publication bias among the 43 included studies used for determining the probability of *Plasmodium* spp. and *Salmonella* spp. co-infection was evaluated using a funnel plot and Egger’s test. The funnel plot exhibited an asymmetrical distribution of ES, and the seES was far from the middle line (no effect) (Supplementary Fig. S5). Egger’s test demonstrated no small study effect (p, 0.379; coefficient, 1.62; standard error, 1.82; t, 0.89). A contour-enhanced funnel plot analysis revealed missing studies in the significant areas (p < 0.01) (Supplementary Fig. S6), indicating that the funnel plot asymmetry was likely due to factors such as heterogeneity, selection bias, and quality of the included studies rather than publication bias.

## Discussion

The present meta-analysis revealed a high prevalence of malaria and typhoidal/NTS co-infections among febrile patients detected using the Widal test (14%) and a low prevalence of malaria and typhoidal/NTS co-infections among febrile patients detected using blood cultures (1%). Moreover, the meta-analysis demonstrated that the prevalence of typhoidal/NTS infection among patients with malaria using the Widal test was high (31%), whereas the prevalence of typhoid/non-typhoid using blood culture was low (3%). A high prevalence of malaria infections among patients with typhoidal *Salmonella* spp. infections (17%) and NTS (43%) was also detected. The highest prevalence of co-infections detected using the Widal test was observed in Cameroon^40^, followed by Nigeria^36,51,59,75,90,103^ and Sierra Leone^87^, compared with Ghana, India, Ethiopia, Tanzania, and Pakistan. In using blood cultures, the gold standard method for the identification of *Salmonella* spp., the results indicated that the highest prevalence of co-infection was reported in Nigeria^37,57,83^ compared with India, Tanzania, Ghana, and Kenya. Based on these results, typhoid/non-typhoid and malaria co-infection among febrile patients frequently occurred in Nigeria. In 2020, Nigeria accounted for the most malaria cases (27%) and malaria-related deaths (23%) worldwide^1^. Moreover, typhoid fever is a major disease in Nigeria due to increased urbanization, insufficient water supply, movement of immigrant workers, poor processing of human waste, and overuse of antibiotics^109^. Due to the co-endemicity of these two pathogens, the possibility of co-infection might increase in this country.

Using the data from studies perfoming blood culture to identify typhoidal/NTS infection, the subgroup analysis of typhoidal/NTS infection demonstrated low prevalence of malaria and typhoid co-infections among febrile patients (1%) and low prevalence of typhoid among patients with malaria (6%). Moreover, the low prevalence of malaria and NTS co-infections among febrile patients (1%) and NTS infection among patients with malaria (2%) was observed. The highest prevalence of malaria and typhoid co-infections among febrile patients was reported in Nigeria, suggesting that malaria and typhoid are indeed halo-endemic in this area^83^. In the meta-analysis of typhoid among patients with malaria, the highest prevalence of typhoid among patients with malaria was noted in Nigeria^38,57^. These results suggested an increasing episode of persistent fever among patients with *S. typhi* and *P. falciparum* infections in Nigeria. For NTS infection among patients with malaria, the prevalence was higest in Kenya, and NTS infection was the most common bacteremia in children with malaria^28^. The high rate of bacteremia in patients with malaria in Nigeria might be due to the high prevalence of NTS infections and malnutrition^28^.

Using the data from studies performing blood culture to identify typhoidal/NTS infection, the subgroup analysis of regions demonstrated that the prevalence of malaria and typhoidal/NTS co-infections were 1% in both Africa and Asia. However, the prevalence of typhoidal/NTS among patients with malaria was higher in Asia (6%) than those of Africa (2%). The difference in the prevalence of typhoidal/NTS co-infections between two regions might be caused by the heterogeneity of the prevalence estimates between two regions or real difference caused by environmental factors. For example, studies in India suggested that malaria and typhoid are endemic because of poor hygiene and environmental factors^104,108^. In Africa, although the pooled prevalence of typhoidal/NTS infection among patients with malaria was lower than those in Asia; the results of individual studies were heterogenous. For example, the high prevalence of typhoidal/NTS infection among patients with malaria were reported by four studies conducted in Nigeria^37,38,57,83^, while a lower prevalence was reported by other studies included in the meta-analysis.

Using the data from studies performing blood culture to identify typhoidal/NTS infection, the subgroup analysis of time (year of publication) showed that the prevalence of malaria and typhoid co-infections among febrile patients, and typhoidal *Salmonella* infections among patients with malaria was highest in 2016, while lower prevalence was reported in before and after 2016. In 2016, three studies conducted in Nigeria and India^66,83,104^ reported the highest prevalence rates of typhoid among patients with malaria. The peak of typhoid among patients with malaria in 2016 was different from those of NTS infections among patients with malaria. The subgroup analysis showed that the peak prevalence rate of NTS infection among patients with malaria was highest in 2011, lower in 2012–2016, and 2001. These results indicated that the prevalence of NTS might decreased with time in 2011–2016, while the prevalence of typhoid among patients with malaria might not depend on time, which are needed to be further investigated.

Using the data from studies performing blood culture to identify typhoidal/NTS infection, the subgroup analysis of age of patients demonstrated that the prevalence rate of typhoidal/NTS infection among patients with malaria was higher in adults (3%) compared to that in children (2%). The previous study showed that peaks of NTS infection occurred in children aged < 2 years and adults aged 25–40 years^110^, while the lower rate of NTS infection occurred in children aged less than 12 years old, and the proportion of hospitalization was decreased with age^111^. These age groups were supported by the subgroup analysis of age that the prevalence of typhoidal/NTS was higher in adults than in children. Nevertheless, as the limitation of age information in studies reported typhoidal/NTS co-infections among febrile patients, the subgroups analysis of age might not represent the exact difference in the prevalence of typhoidal/NTS co-infections between adults and children.

The present meta-analysis demonstrated a wide gap in prevalence of malaria and typhoid/non-typhoid co-infections among febrile patients as measured by the Widal test and blood culture in analysis. The high rate of typhoid/non-typhoid and malaria co-infections detected using the Widal test and low rate of co-infections detected using blood cultures might be due to the lack of differentiation between *Salmonella* species/serotypes by the Widal test and cross-reactivity with other Enterobacteriaceae^5,7^. Moreover, false-positive Widal tests have been reported in patients with malaria and other infections^5^. The malaria *Plasmodium* may share similar strong immunogenic antigens with the typhoidal *Salmonella* (*S. typhi*); thus, *Plasmodium* infections could induce the generation of antibodies against *S. typhi* antigens, leading to cross-reactivity and false-positive results^47^. Furthermore, malaria loading strongly correlated with *Salmonella* antibody titers in numerous studies^47^. This cross-reaction of typhoidal/NTS antibodies with malarial antigen leads to overdiagnosis of typhoid fever^74,112^. The Widal test also generates false-negative results if patients are tested during the early phase of typhoid fever^5^. The high prevalence of typhoid fever may also be due to poor interpretation of the Widal test when diagnosing typhoid fever^113^. Nevertheless, in Africa and other territories, the Widal test is the most common diagnostic tool used for typhoid fever; owing to its low cost, ease of performance, and minimal training and equipment requirements. Of note, false-positive results of the Widal tests in febrile patients suspected of having *Salmonella* spp. infection may lead to incorrect treatment for malaria parasites. Thus, careful interpretation of the Widal test for the diagnoses of typhoid fever in resource-poor countries is required, as the overdiagnosis of typhoid fever can lead to unnecessary treatment of patients with antibiotics, microbial resistance, and poor outcome. The use of Widal test alone for the diagnosis of typhoid fever will cause misdiagnoses.

Using blood cultures alone to identify *Salmonella* spp. infection may underestimate *Salmonella* spp. infections, as blood culture has a lower sensitivity compared with the Widal test. Negative blood culture test results may be noted in patients with acute disease before the antibody response^5^. Based on the results of this study, the Widal test should not be used alone but in combination with blood/stool cultures. Therefore, a combination of the Widal test and blood and stool cultures is an excellent choice for diagnosing *Salmonella* spp. infection among febrile patients or patients with malaria. Although the high laboratory expenses for combination testing are difficult to overcome, the use of more than one diagnostic method to identify *Salmonella* spp. infections among patients with malaria is important to prevent incorrect treatment and misdiagnoses of malaria and other acute febrile illnesses. Infections caused by typhoidal *Salmonella*, including *S. typhi* and *S. paratyphi*, and the associated serious complications require treatment with antibiotics, including chloramphenicol, cefixime, amoxicillin, trimethoprim/sulfamethoxazole, azithromycin, aztreonam, and cefotaxime, to prevent severe illness and death^3,114^. NTS infections do not usually require treatment with antibiotics. However, complications, such as septicemia and meningitis, require treatment with ciprofloxacin, ceftriaxone, and ampicillin, according to the WHO^3,114^. Presently, antibiotic resistance of *Salmonella* species is an emerging threat, so reliable diagnostic test and appropriate treatments for typhoid/non-typhoid fever are important.

The present meta-analysis demonstrated that *Salmonella* spp. bacteremia developed in approximately 2% of patients with severe malaria. This occurrence was not much different from the *Salmonella* spp. bacteremia pooled prevalence of 3% in patients with non-severe malaria. Several mechanisms have been suggested to elucidate why patients with malaria may be predisposed to *Salmonella* spp. infection and bacteremia. First, immunosuppression occurs during malaria infection and treatment^115^. Second, malaria can lead to hemolysis, which may predispose patients to infection with Gram-negative bacteria, such as typhoidal *Salmonella*/NTS spp.^69^. Third, changes in iron storage metabolism from malaria-induced hemolysis cause neutrophil dysfunction and increased susceptibility^116–118^. Increased free iron from hemolysis may also promote the survival of *Salmonella* spp.^19^. Fourth, the sequestration of parasitized red blood cells in the intestine causes reduced blood flow in the mucosal gut barrier, which increases intestinal susceptibility to bacterial infection^119,120^. The high rate of NTS bacteremia is well described in patients with malaria-related severe anemia^121^. Severe anemia and hemolysis increase the iron level in the blood and tissues; therefore, pathogens can be actively transported, and iron acquisition is easier^121^. Based on our results, the increased risk of typhoidal *Salmonella* bacteremia in patients with severe malaria might reflect the high rate of parasite sequestration and vital organ dysfunction. Moreover, bacteremia cannot be excluded from patients with severe malaria; severe malaria is difficult to distinguish from bacterial sepsis^56,85^. Therefore, the WHO guidelines for malaria recommend that children with severe falciparum malaria in high-transmission areas should receive empirical broad-spectrum antibacterial therapy. However, empirical antibiotics should not be administered to adults with severe malaria unless there is clear evidence of bacterial infection^122^. In the low-transmission areas, WHO suggests that physicians should determine whether patients should receive antibiotics depending on the patient’s condition or parasitemia levels, but patients with severe malaria should not be routinely treated with antibiotics^122,123^. In addition to the WHO guidelines, two studies conducted in Myanmar^58,78^ stated that “clinicians should have a lower threshold for commencing empirical antibacterial therapy in adults diagnosed with falciparum malaria in these locations than is presently recommended.”

The present meta-analysis revealed that typhoidal/NTS and malaria co-infection occurred by chance when the random-effects model was employed to combine the effect estimates. However, the subgroup analysis demonstrated a decreased odds ratio of co-infection in children aged < 15 years, indicating that the current malaria infection was negatively associated with typhoidal/non-typhoidal *Salmonella* spp. infection in children^35,41,44,46,53,73^. Although the meta-analysis did not provide a significant outcome, individual studies demonstrated significantly higher odds ratios of co-infection in Nigeria^57,59,82,90,95,97,101^, Cameroon^40^, India^104^, and Pakistan^102^ and significantly lower odds ratio of co-infection in Nigeria^36,47,51^, Cameroon^73,92,93^, Pakistan^52,70^, Ghana^41,53^, Tanzania^44^, Kenya^60^, and India^43,71^. Based on these results, the probability of co-infection varies. The fixed-effects model indicated that typhoid/non-typhoid and malaria co-infection did not occur by chance or that there was an association between typhoid/non-typhoid and malaria co-infection in some way. Further studies are required to investigate this association.

The present meta-analysis of case fatality rate of patients with co-infection demonstrated the high rate of mortality (16%) without heterogeneity among the three included studies^60,80,92^. These three studies enrolled patients with severe malaria and co-infected with NTS and indicated that both diseases facilitate the higher fatality rate than those of the malaria or NTS infection alone. Moreover, the meta-analysis of two studies^79,80^ showed a higher mean parasitemia level in patients with malaria and co-infected with NTS compared to those with malaria alone (without heterogeneity, 0%), but it is important to note the limitation in the number of included studies in the analysis. Therefore, there is a need to investigate if co-infection of malaria and NTS leads to poor outcome or demonstrated the association of both diseases.

This study had several limitations. First, most included studies were cross-sectional studies that determined the prevalence of typhoidal/non-typhoidal *Salmonella* spp. and malaria co-infection. Therefore, data were not available to determine the differences between co-infected patients and mono-infected patients. Second, the number of studies evaluating the occurrence of *Salmonella* spp. bacteremia in patients with severe malaria was limited; therefore, the pooled prevalence of *Salmonella* spp. bacteremia in patients with severe malaria might not represent all patients with severe malaria. Third, the heterogeneity among the included studies used to determine the probability of typhoidal/non-typhoidal *Salmonella* spp. and malaria co-infection was high; therefore, the association between typhoidal/non-typhoidal *Salmonella* spp. and malaria co-infection should be carefully interpreted with the results from the sensitivity test. Compared with the previous systematic review^32^, the present study excluded studies with recent malaria infection; most included studies used microscopy rather than RDTs for malaria detection; and there was no publication bias among the included studies.

In conclusion, whether typhoidal/non-typhoidal *Salmonella* spp. and malaria co-infection occurred by chance or not, healthcare providers must provide support to patients with nonspecific clinical symptoms of malaria or typhoidal/non-typhoidal diseases. In the present study, malaria associated with typhoidal/NTS infection in children and the high case fatality rate among few co-infected patients were highlighted. Future prospective longitudinal studies using the appropriate and confirmatory diagnosis for *Salmonella* spp. infections are highly recommended to ensure the real prevalence of co-infection and highlight the outcome of co-infection for providing adequate treatment of co-infections in febrile patients who live in areas where malaria is endemic like tropical Africa or India.

## Supplementary Information

Supplementary Figure S1.

Supplementary Figure S2.

Supplementary Figure S3.

Supplementary Figure S4.

Supplementary Figure S5.

Supplementary Figure S6.

Supplementary Table S1.

Supplementary Table S2.

## Data Availability

All data related to the present study in this manuscript are available.

## References

[1] WHO (2020). World Malaria Report 2020.

[2] Kuhn KG, Falkenhorst G, Ceper TH, Dalby T, Ethelberg S, Molbak K (2012). Detecting non-typhoid Salmonella in humans by ELISAs: A literature review. J. Med. Microbiol..

[3] Smith SI, Seriki A, Ajayi A (2016). Typhoidal and non-typhoidal Salmonella infections in Africa. Eur. J. Clin. Microbiol. Infect. Dis..

[4] Buckle GC, Walker CL, Black RE (2012). Typhoid fever and paratyphoid fever: Systematic review to estimate global morbidity and mortality for 2010. J. Glob. Health.

[5] Deksissa T, Gebremedhin EZ (2019). A cross-sectional study of enteric fever among febrile patients at Ambo hospital: Prevalence, risk factors, comparison of Widal test and stool culture and antimicrobials susceptibility pattern of isolates. BMC Infect. Dis..

[6] Azmatullah A, Qamar FN, Thaver D, Zaidi AK, Bhutta ZA (2015). Systematic review of the global epidemiology, clinical and laboratory profile of enteric fever. J. Glob. Health..

[7] Gut AM, Vasiljevic T, Yeager T, Donkor ON (2018). Salmonella infection—Prevention and treatment by antibiotics and probiotic yeasts: A review. Microbiology (Reading).

[8] Andino A, Hanning I (2015). *Salmonella enterica*: Survival, colonization, and virulence differences among serovars. Sci. World J..

[9] Andrews JR, Ryan ET (2015). Diagnostics for invasive Salmonella infections: Current challenges and future directions. Vaccine..

[10] Sanchez-Vargas FM, Abu-El-Haija MA, Gomez-Duarte OG (2011). Salmonella infections: An update on epidemiology, management, and prevention. Travel Med. Infect. Dis..

[11] Ford L, Glass K, Veitch M, Wardell R, Polkinghorne B, Dobbins T (2016). Increasing incidence of Salmonella in Australia, 2000–2013. PLoS ONE.

[12] Crump JA, Sjolund-Karlsson M, Gordon MA, Parry CM (2015). Epidemiology, clinical presentation, laboratory diagnosis, antimicrobial resistance, and antimicrobial management of invasive salmonella infections. Clin. Microbiol. Rev..

[13] Bula-Rudas FJ, Rathore MH, Maraqa NF (2015). Salmonella infections in childhood. Adv. Pediatr..

[14] Barrett FC, Knudsen JD, Johansen IS (2013). Cases of typhoid fever in Copenhagen region: A retrospective study of presentation and relapse. BMC Res Notes.

[15] Bennett SD, Lowther SA, Chingoli F, Chilima B, Kabuluzi S, Ayers TL (2018). Assessment of water, sanitation and hygiene interventions in response to an outbreak of typhoid fever in Neno District, Malawi. PLoS ONE.

[16] Brockett S, Wolfe MK, Hamot A, Appiah GD, Mintz ED, Lantagne D (2020). Associations among water, sanitation, and hygiene, and food exposures and typhoid fever in case-control studies: A systematic review and meta-analysis. Am. J. Trop. Med. Hyg..

[17] Graham SM, Walsh AL, Molyneux EM, Phiri AJ, Molyneux ME (2000). Clinical presentation of non-typhoidal *Salmonella bacteraemia* in Malawian children. Trans. R. Soc. Trop. Med. Hyg..

[18] Mweu E, English M (2008). Typhoid fever in children in Africa. Trop. Med. Int. Health.

[19] Morpeth SC, Ramadhani HO, Crump JA (2009). Invasive non-Typhi Salmonella disease in Africa. Clin. Infect. Dis..

[20] Mtove G, Amos B, von Seidlein L, Hendriksen I, Mwambuli A, Kimera J (2010). Invasive salmonellosis among children admitted to a rural Tanzanian hospital and a comparison with previous studies. PLoS ONE.

[21] Siba V, Horwood PF, Vanuga K, Wapling J, Sehuko R, Siba PM (2012). Evaluation of serological diagnostic tests for typhoid fever in Papua New Guinea using a composite reference standard. Clin. Vaccine Immunol..

[22] Kumar P, Kumar R (2017). Enteric fever. Indian J. Pediatr..

[23] Wain J, Pham VB, Ha V, Nguyen NM, To SD, Walsh AL (2001). Quantitation of bacteria in bone marrow from patients with typhoid fever: Relationship between counts and clinical features. J. Clin. Microbiol..

[24] Wain J, Diep TS, Bay PV, Walsh AL, Vinh H, Duong NM (2008). Specimens and culture media for the laboratory diagnosis of typhoid fever. J. Infect. Dev. Ctries..

[25] Beyene G, Asrat D, Mengistu Y, Aseffa A, Wain J (2008). Typhoid fever in ethiopia. J. Infect. Dev. Ctries.

[26] Prabagaran SR, Kalaiselvi V, Chandramouleeswaran N, Deepthi KNG, Brahmadathan KN, Mani M (2017). Molecular diagnosis of *Salmonella typhi* and its virulence in suspected typhoid blood samples through nested multiplex PCR. J. Microbiol. Methods.

[27] Walsh AL, Phiri AJ, Graham SM, Molyneux EM, Molyneux ME (2000). Bacteremia in febrile Malawian children: Clinical and microbiologic features. Pediatr. Infect. Dis. J..

[28] Were T, Davenport GC, Hittner JB, Ouma C, Vulule JM, Ong'echa JM (2011). Bacteremia in Kenyan children presenting with malaria. J. Clin. Microbiol..

[29] Brent AJ, Oundo JO, Mwangi I, Ochola L, Lowe B, Berkley JA (2006). *Salmonella bacteremia* in Kenyan children. Pediatr. Infect. Dis. J..

[30] Bronzan RN, Taylor TE, Mwenechanya J, Tembo M, Kayira K, Bwanaisa L (2007). Bacteremia in Malawian children with severe malaria: Prevalence, etiology, HIV coinfection, and outcome. J. Infect. Dis..

[31] Mabey DC, Brown A, Greenwood BM (1987). *Plasmodium falciparum* malaria and Salmonella infections in Gambian children. J. Infect. Dis..

[32] Church J, Maitland K (2014). Invasive bacterial co-infection in African children with Plasmodium falciparum malaria: A systematic review. BMC Med..

[33] Moher D, Liberati A, Tetzlaff J, Altman DG, PRISMA Group (2009). Preferred reporting items for systematic reviews and meta-analyses: the PRISMA statement. PLoS Med..

[34] Moola SMZ, Tufanaru C, Aromataris E, Sears K, Sfetcu R, Currie M, Qureshi R, Mattis P, Lisy K, Mu P-F (2020). Chapter 7: Systematic Reviews of Etiology and Risk.

[35] Achonduh-Atijegbe OA, Mfuh KO, Mbange AH, Chedjou JP, Taylor DW, Nerurkar VR (2016). Prevalence of malaria, typhoid, toxoplasmosis and rubella among febrile children in Cameroon. BMC Infect. Dis..

[36] Agwu E, Ihongbe JC, Okogun GR, Inyang NJ (2009). High incidence of co-infection with Malaria and Typhoid in febrile HIV infected and AIDS patients in Ekpoma, Edo State, Nigeria. Braz. J. Microbiol..

[37] Akinyemi KO, Bamiro BS, Coker AO (2007). Salmonellosis in Lagos, Nigeria: Incidence of *Plasmodium falciparum*-associated co-infection, patterns of antimicrobial resistance, and emergence of reduced susceptibility to fluoroquinolones. J. Health Popul. Nutr..

[38] Akinyemi KO, Iwalokun BA, Alafe OO, Mudashiru SA, Fakorede C (2015). bla CTX-M-I group extended spectrum beta lactamase-producing *Salmonella typhi* from hospitalized patients in Lagos. Nigeria. Infect. Drug Resist..

[39] Ali MA, James OC, Mohamed AA, Joachim A, Mubi M, Omodior O (2020). Etiologic agents of fever of unknown origin among patients attending Mnazi Mmoja Hospital, Zanzibar. J. Community Health.

[40] Ammah A, Nkuo-Akenji T, Ndip R, Deas JE (1999). An update on concurrent malaria and typhoid fever in Cameroon. Trans. R. Soc. Trop. Med. Hyg..

[41] Anabire NG, Aryee PA, Addo F, Anaba F, Kanwugu ON, Ankrah J (2018). Evaluation of hematological indices of childhood illnesses in Tamale Metropolis of Ghana. J. Clin. Lab. Anal..

[42] Anjorin AA, Nwammadu JE (2020). Seroepidemiology of seasonal influenza virus among unvaccinated pregnant women in Lagos, Nigeria. Infez. Med..

[43] Bhalla K, Rao P, Manipura R (2019). A clinico-epidemiological analysis of seropositive cases of tropical infections and their co-infection in tertiary care hospital in South India. Indian J. Public Health Res. Dev..

[44] Biggs HM, Lester R, Nadjm B, Mtove G, Todd JE, Kinabo GD (2014). Invasive Salmonella infections in areas of high and low malaria transmission intensity in Tanzania. Clin. Infect. Dis..

[45] Birhanie M, Tessema B, Ferede G, Endris M, Enawgaw B (2014). Malaria, typhoid fever, and their coinfection among febrile patients at a rural health center in Northwest Ethiopia: A cross-sectional study. Adv. Med..

[46] Chipwaza B, Mhamphi GG, Ngatunga SD, Selemani M, Amuri M, Mugasa JP (2015). Prevalence of bacterial febrile illnesses in children in Kilosa district, Tanzania. PLoS Negl. Trop. Dis..

[47] Enabulele O, Awunor SN (2016). Typhoid fever in a Tertiary Hospital in Nigeria: Another look at the Widal agglutination test as a preferred option for diagnosis. Niger. Med. J..

[48] Eze EA, Ukwah BN, Okafor PC, Ugwu KO (2011). Prevalence of malaria and typhoid co-infections in University of Nigeria, Nsukka District of Enugu State. Nigeria. Afr. J. Biotechnol..

[49] Falay D, Kuijpers LMF, Phoba MF, De Boeck H, Lunguya O, Vakaniaki E (2016). Microbiological, clinical and molecular findings of non-typhoidal Salmonella bloodstream infections associated with malaria, Oriental Province, Democratic Republic of the Congo. BMC Infect. Dis..

[50] Igbeneghu C, Olisekodiaka MJ, Onuegbu JA (2009). Malaria and typhoid fever among adult patients presenting with fever in Ibadan, southwest Nigeria. Int. J. Trop. Med..

[51] Igharo EA, Osazuwa F, Ajayi SA, Ebueku A, Igbinigie O (2012). Dual infection with typhoid and malaria in febrile patients in Ikare Akoko, Nigeria. Int. J. Trop. Med..

[52] Jalani HA, Shah SMA, Anjum F, Khan SG, Akhter N, Khan M (2019). Prevalence and co-infection of Malaria and Typhoid in the local population of Faisalabad, Pakistan. Pak. J. Pharm. Sci..

[53] Krumkamp R, Kreuels B, Sarpong N, Boahen KG, Foli G, Hogan B (2016). Association between malaria and invasive nontyphoidal Salmonella infection in a hospital study: Accounting for Berkson's bias. Clin. Infect. Dis..

[54] Maltha J, Guiraud I, Kaboré B, Lompo P, Ley B, Bottieau E (2014). Frequency of severe malaria and invasive bacterial infections among children admitted to a rural hospital in Burkina Faso. PLoS ONE.

[55] Mourembou G, Nzondo SM, Ndjoyi-Mbiguino A, Lekana-Douki JB, Kouna LC, Matsiegui PB (2016). Co-circulation of Plasmodium and bacterial DNAs in blood of febrile and afebrile children from urban and rural areas in Gabon. Am. J. Trop. Med. Hyg..

[56] Nielsen MV, Amemasor S, Agyekum A, Loag W, Marks F, Sarpong N (2015). Clinical indicators for bacterial co-infection in Ghanaian children with *P*. *falciparum* infection. PLoS ONE.

[57] Nwuzo AC, Onyeagba RA, Iroha IR, Nworie O, Oji AE (2009). Parasitological, bacteriological, and cultural determination of prevalence of malaria parasite (*Plasmodium falciparum*) and typhoid fever co-infection in Abakaliki, Ebonyi State. Sci. Res. Essays.

[58] Nyein PP, Aung NM, Kyi TT, Htet ZW, Anstey NM, Kyi MM (2016). High frequency of clinically significant bacteremia in adults hospitalized with falciparum malaria. Open Forum Infect. Dis..

[59] Odikamnoro OO, Ikeh IM, Okoh FN, Ebiriekwe SC, Nnadozie IA, Nkwuda JO (2018). Incidence of malaria/typhoid co-infection among adult population in Unwana community, Afikpo north local government area, Ebonyi State, Southeastern Nigeria. Afr. J. Infect. Dis..

[60] Oundo JO, Muli F, Kariuki S, Waiyaki PG, Iijima Y, Berkley J (2002). Non-typhi salmonella in children with severe malaria. E. Afr. Med. J..

[61] Pam VA, Landan S, Adejoh VA, Pam DD, Danjuma K (2018). Co-infection of malaria and typhoid fever among pregnant women attending antenatal clinics at general hospital, wuse, federal capital territory (FCT), Abuja, Nigeria. Niger. J. Parasitol..

[62] Park SE, Pak GD, Aaby P, Adu-Sarkodie Y, Ali M, Aseffa A (2016). The relationship between invasive nontyphoidal Salmonella disease, other bacterial bloodstream infections, and malaria in Sub-Saharan Africa. Clin. Infect. Dis..

[63] Phu NH, Day NPJ, Tuan PQ, Mai NTH, Chau TTH, Van Chuong L (2020). Concomitant bacteremia in adults with severe falciparum malaria. Clin. Infect. Dis..

[64] Popoola O, Kehinde A, Ogunleye V, Adewusi OJ, Toy T, Mogeni OD (2019). Bacteremia among febrile patients attending selected healthcare facilities in Ibadan, Nigeria. Clin. Infect. Dis..

[65] Qureshi AW, Khan ZU, Khan L, Mansoor A, Minhas R (2019). Prevalence of malaria, typhoid and co-infection in district dir (lower), Pakistan. Biosci. J..

[66] Raja JM, Mary A, Usha S (2016). A study on dual infections in pyrexia cases. Int. J. Med. Res. Health Sci..

[67] Ramya TG, Sunitha BR (2017). Enteric fever cases showing concurrent seropositivity with dengue and malaria: A sero-diagnostic challenge. Microbiol. Res..

[68] Samatha P, Rao KC, Sowmya BS (2015). Malaria typhoid co-infection among febrile patients. J. Evol. Med. Dent. Sci..

[69] Sandlund J, Naucler P, Dashti S, Shokri A, Eriksson S, Hjertqvist M (2013). Bacterial coinfections in travelers with malaria: Rationale for antibiotic therapy. J. Clin. Microbiol..

[70] Shaikh S, Jamali GM, Jamali AA, Tanwani BM, Malik AS, Shaikh NS (2018). Malaria and typhoid fever: positive Widal test in malaria patients reported at tertiary care hospital. Indo Am. J. Pharm. Sci..

[71] Singh R, Singh SP, Ahmad N (2014). A study of etiological pattern in an epidemic of acute febrile illness during monsoon in a tertiary health care institute of Uttarakhand, India. J. Clin. Diagn. Res..

[72] Tabu C, Breiman RF, Ochieng B, Aura B, Cosmas L, Audi A (2012). Differing burden and epidemiology of non-Typhi Salmonella bacteremia in rural and urban Kenya, 2006–2009. PLoS ONE.

[73] Tchuandom SB, Tchouangueu TF, Antonio-Nkondjio C, Lissom A, Djang JON, Atabonkeng EP (2018). Seroprevalence of dengue virus among children presenting with febrile illness in some public health facilities in Cameroon. Pan Afr. Med. J..

[74] Verma D, Kishore S, Siddique ME (2014). Comparative evaluation of various tests for diagnosis of concurrent malaria and typhoid fever in a tertiary care hospital of northern India. J. Clin. Diagn. Res..

[75] Abah AEFP (2019). Preliminary investigation of malaria, typhoid and their coinfection among febrile subjects in Port Harcourt, Rivers State Nigeria. Eur. J. Pharm. Med. Res..

[76] Afoakwah RAD, Boampong J, Baidoo M, Nwaefuna E, Tefe P (2011). Typhoid malaria coinfection in Ghana. Eur. J. Exp. Biol..

[77] Alhassan HSN, Manga S, Abdullahi K, Hamid K (2012). Co-infection profile of *Salmonella typhi* and malaria parasite in Sokoto-Nigeria. Int. J. Eng. Sci. Technol..

[78] Aung NM, Nyein PP, Htut TY, Htet ZW, Kyi TT, Anstey NM (2018). Antibiotic therapy in adults with malaria (ANTHEM): High rate of clinically significant bacteremia in hospitalized adults diagnosed with falciparum malaria. Am. J. Trop. Med. Hyg..

[79] Bassat Q, Guinovart C, Sigauque B, Mandomando I, Aide P, Sacarlal J (2009). Severe malaria and concomitant bacteraemia in children admitted to a rural Mozambican hospital. Trop. Med. Int. Health.

[80] Berkley J, Mwarumba S, Bramham K, Lowe B, Marsh K (1999). Bacteraemia complicating severe malaria in children. Trans. R. Soc. Trop. Med. Hyg..

[81] Bhattacharya SK, Sur D, Dutta S, Kanungo S, Ochiai RL, Kim DR (2013). Vivax malaria and bacteraemia: A prospective study in Kolkata, India. Malaria J..

[82] Chukwuma OG, Taiwo SO, Adekeye BT (2014). Prevalence of Plasmodium and Salmonella infections among pregnant women with fever, presented to three hospitals in Ogun and Lagos State, South-West Nigeria. Int. Blood Res. Rev..

[83] Edet UOER, Etok CA, Ukanukumo JA (2016). Prevalence of malaria and typhoid co-infection amongst residents of Uyo, Akwa Ibom State. Nigeria. Int. J. Infect. Dis..

[84] Ekesiobi AOIM, Njoku OO (2017). Co-infection of malaria and typhoid fever in a tropical community. Anim. Res. Int..

[85] Evans JA, Adusei A, Timmann C, May J, Mack D, Agbenyega T (2004). High mortality of infant bacteraemia clinically indistinguishable from severe malaria. QJM.

[86] Ibrahim SYS, Wenceslas BW, Constant S, Fabrice D, Soufiane DS, Jacques Z, Armel P, Salam OA, Sanata B (2019). Malaria and typhoid fever coinfection in the Hospital University of Bobo-Dioulasso, Burkina Faso. J. Parasitol. Res..

[87] Kargbo MSML, Samura SK, Meng X, Zou F (2014). The relative prevalence of typhoid and malaria in febrile patients in Freetown, Sierra Leone. Open J. Prev. Med..

[88] Katiyar GDAA, Khan S, Chaudhary BC, Sharma M (2020). Malaria or typhoid co-infection in a tertiary care hospital of Bareilly, Uttar Pradesh, India. Int. J. Community Med. Public Health.

[89] Mbuh FAGM, Ogbadu L (2003). Rate of coinfection with malaria parasites and *Salmonella typhi* in Zaria, Kaduna State, Nigeria. Ann. Afr. Med..

[90] Mike IML, Adamu A, Longshit SG, Garba M, Sado F, Shittu AN (2017). Prevalence of malaria and typhoid co-infections among patients who attended State Specialist Hospital Gombe from May to August 2015 for malaria and Widal tests. Greener J. Epidemiol. Public Health.

[91] Mohammed HIMI, Sadiq HA (2020). Malaria and typhoid fever: Prevalence, co-infection and socio-demographic determinants among pregnant women attending antenatal care at a primary healthcare facility in Central Nigeria. Int. J. Pathog. Res..

[92] Ndip LMEF, Kimbi HK, Njom HA, Ndip RN (2015). Co-infection of malaria and typhoid fever in feverish patients in the Kumba Health District, Southwest Cameroon: Public health implications. Int J. Trop. Dis. Health.

[93] Njolle ABTB, Asaah S, Forfuet DF, Kamga HLF (2020). The prevalence of Salmonellosis in patients with malaria attending an urban hospital in Douala, Littoral region, Cameroon. J. Adv. Med. Med. Res..

[94] Nwabueze USCO-OA (2013). Rate of malaria-typhoid co-infection among pregnant women attending antenatal clinics in Anambra State South-east Nigeria. Int. J. Trop. Med. Public Health.

[95] Ohanu ME, Mbah AU, Okonkwo PO, Nwagbo FS (2003). Interference by malaria in the diagnosis of typhoid using Widal test alone. W. Afr. J. Med..

[96] Omoya FOAO (2017). Co-infection of malaria and typhoid fever among pregnant women attending primary health care centre, Ojo Local Government, Lagos, Nigeria. Health Sci. J..

[97] Onyido AEIC, Umeanaeto PU, Irikannu KC, Aribodor DN, Ezeanya LC, Ugha CN, Obiechina IO (2014). Co-Infection of malaria and typhoid fever in Ekwulumili Community Anambra State, South-east Nigeria. N. Y. Sci. J..

[98] Orok DUA, Ikpan O, Duke E, Eyo E, Edadi U, Ati B, Udida J (2016). Prevalence of malaria and typhoid fever co-infection among febrile patients attending College of Health Technology Medical Centre in Calabar, Cross River State Nigeria. Niger. Int. J. Curr. Microbiol. Appl. Sci..

[99] Oshiokhayamhe IKNO, Israel IO, Agumeile K-I (2021). Assessment of the prevalence of malaria and typhoid fever among apparently healthy undergraduates. Int. J. Med. Sci. Public Health.

[100] Ozumba GI, Adejoh VA, Danjuma K (2020). Co-infection of malaria and typhoid fever among pregnant women attending antenatal clinics at Dalhatu Araf Specialist Hospital Lafia, Nasarawa State, Nigeria. J. Infect. Dis. Prev. Med.

[101] Pam VALS, Pamc DD, Gullekd JF, Okorof J, Ogbue KI, Bote CJ, Akinyerae AO (2015). The prevalence of malaria and typhoid co-infection in pregnant women attending antenatal in Wuse general hospital Abuja, Nigeria. J. Vet. Adv..

[102] Sajid MZM, Ahmad R, Rasool M, Shah M, Ullah I, Jawad SM (2017). Co infection of malaria and typhoid in district Dir (Lower) Khyber Pakhtunkhwa, Pakistan. J. Entomol. Zool. Stud..

[103] Sale MPM, Adedeji BAM, Shehu A (2020). Prevalence of typhoid and malaria co-infection among patients attending a public hospital in Yola, Nigeria. Int. J. Mosq. Res..

[104] Sharma BMM, Gaind R, Pandey K (2016). Malaria and typhoid co-infection in India: A diagnostic difficulty. J. Dent. Med. Sci..

[105] Snehanshu SHP, Chandrim S, Parul C, Chaudhary B (2014). Malaria and typhoid, do they coexist as an alternative diagnosis in tropics? A tertiary care hospital experience. Int. J. Curr. Microbiol. Appl. Sci..

[106] Sur D, von Seidlein L, Manna B, Dutta S, Deb AK, Sarkar BL (2006). The malaria and typhoid fever burden in the slums of Kolkata, India: Data from a prospective community-based study. Trans. R. Soc. Trop. Med. Hyg..

[107] Ukaegbu CONA, Mawak JD, Igwe CC (2014). Incidence of concurrent malaria and typhoid fever infection in febrile patients in Jos, Plateau State Nigeria. Int. J. Sci. Technol. Res..

[108] Vats AD (2018). Incidence of co-infection of malaria and typhoid and their diagnostic dilemmas. RAPL.

[109] Akinyemi KO, Oyefolu AOB, Mutiu WB, Iwalokun BA, Ayeni ES, Ajose SO (2018). Typhoid fever: Tracking the trend in Nigeria. Am. J. Trop. Med. Hyg..

[110] Gordon MA (2011). Invasive nontyphoidal Salmonella disease: Epidemiology, pathogenesis and diagnosis. Curr. Opin. Infect. Dis..

[111] Saha S, Islam MS, Sajib MSI, Saha S, Uddin MJ, Hooda Y (2019). Epidemiology of typhoid and paratyphoid: Implications for vaccine policy. Clin. Infect. Dis..

[112] Sundufu AJJM, Foday IK (2012). Role of co-infection with malaria parasites and Salmonella typhoid in Bo City, Southern Sierra Leone. Public Health Res..

[113] Sultana S, Hossain MA, Paul SK, Kabir MR, Yesmin T, Maruf MA (2014). Evaluation of TH agglutinin titres of Widal test in the diagnosis of typhoid fever. Mymensingh Med. J..

[114] Parry CM, Hien TT, Dougan G, White NJ, Farrar JJ (2002). Typhoid fever. N. Engl. J. Med..

[115] Alemu A, Shiferaw Y, Addis Z, Mathewos B, Birhan W (2013). Effect of malaria on HIV/AIDS transmission and progression. Parasit. Vectors.

[116] Cunnington AJ, de Souza JB, Walther M, Riley EM (2011). Malaria impairs resistance to Salmonella through heme- and heme oxygenase-dependent dysfunctional granulocyte mobilization. Nat. Med..

[117] Cunnington AJ, Njie M, Correa S, Takem EN, Riley EM, Walther M (2012). Prolonged neutrophil dysfunction after *Plasmodium falciparum* malaria is related to hemolysis and heme oxygenase-1 induction. J. Immunol. (Baltimore).

[118] Roux CM, Butler BP, Chau JY, Paixao TA, Cheung KW, Santos RL (2010). Both hemolytic anemia and malaria parasite-specific factors increase susceptibility to nontyphoidal *Salmonella enterica* serovar typhimurium infection in mice. Infect. Immun..

[119] Seydel KB, Milner DA, Kamiza SB, Molyneux ME, Taylor TE (2006). The distribution and intensity of parasite sequestration in comatose Malawian children. J. Infect. Dis..

[120] Olsson RA, Johnston EH (1969). Histopathologic changes and small-bowel absorption in falciparum malaria. Am. J. Trop. Med. Hyg..

[121] Abuga KM, Muriuki JM, Williams TN, Atkinson SH (2020). How severe anaemia might influence the risk of invasive bacterial infections in African children. Int. J. Mol. Sci..

[122] WHO (2021). WHO Guidelines for Malaria.

[123] White, N. J. Reply to Aung et al. Clinical infectious diseases: An official publication of the Infectious Diseases Society of America **72**(3), 536–538 (2021). 10.1093/cid/ciaa733 PMC7850525 33527125

