# Supplementary figures and images for: Prevalence, probability, and outcomes of typhoidal/non-typhoidal Salmonella and malaria co-infection among febrile patients: a systematic review and meta-analysis
Source: Sci Rep. 2021 Nov 8;11:21889. doi: 10.1038/s41598-021-00611-0 (PMC8576030; doi:10.1038/s41598-021-00611-0)

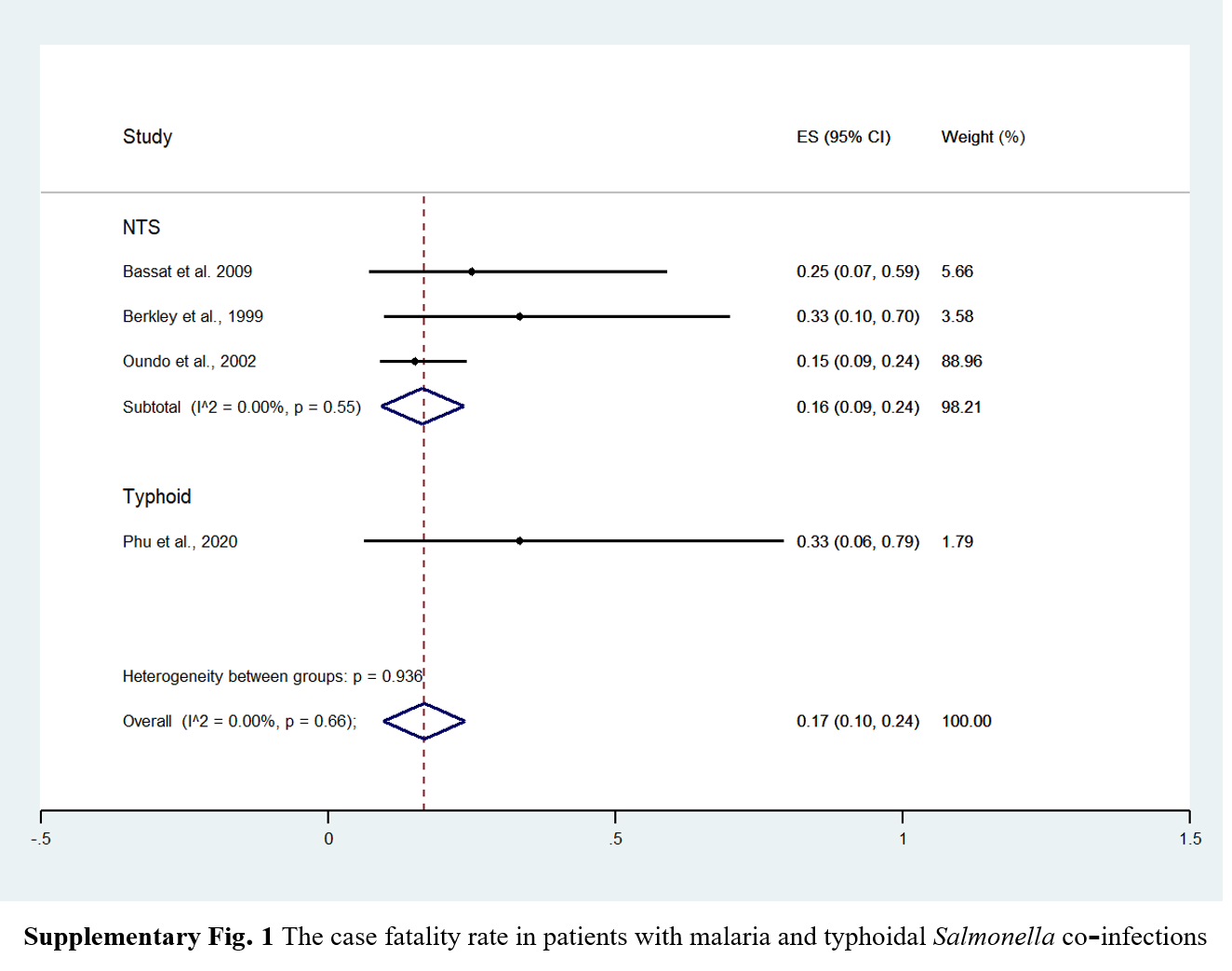

Supplement: Supplementary file 1 — Supplementary Figure S1. [file 41598_2021_611_MOESM1_ESM.png]

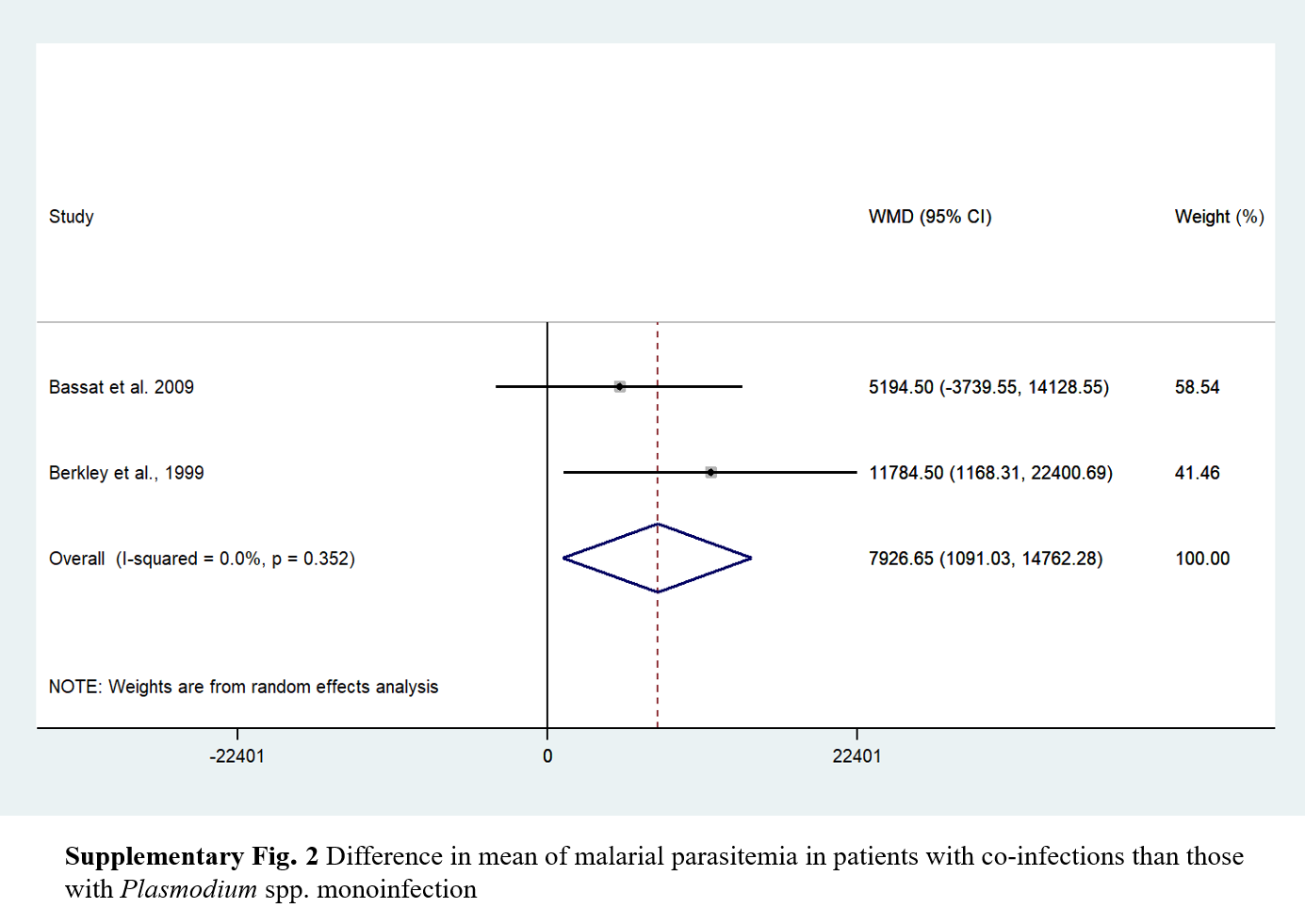

Supplement: Supplementary file 2 — Supplementary Figure S2. [file 41598_2021_611_MOESM2_ESM.png]

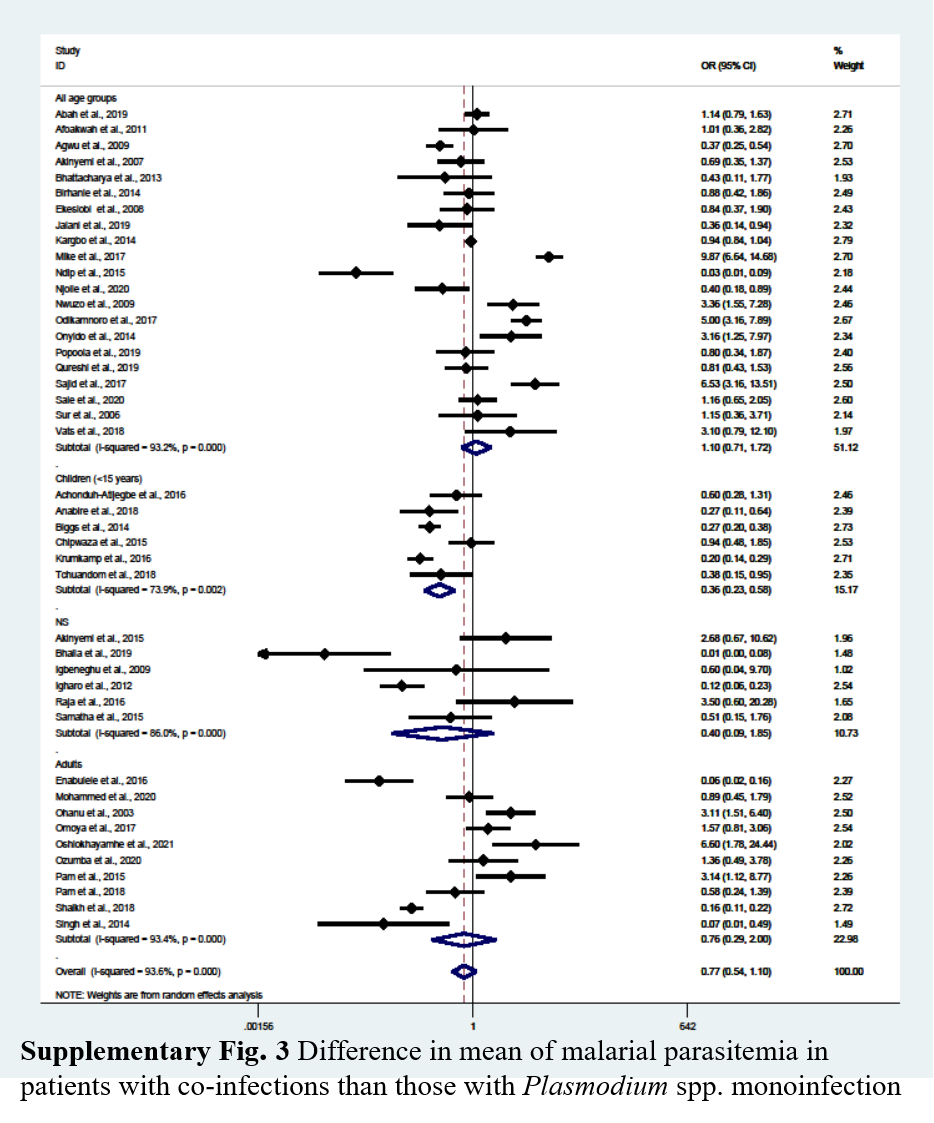

Supplement: Supplementary file 3 — Supplementary Figure S3. [file 41598_2021_611_MOESM3_ESM.png]

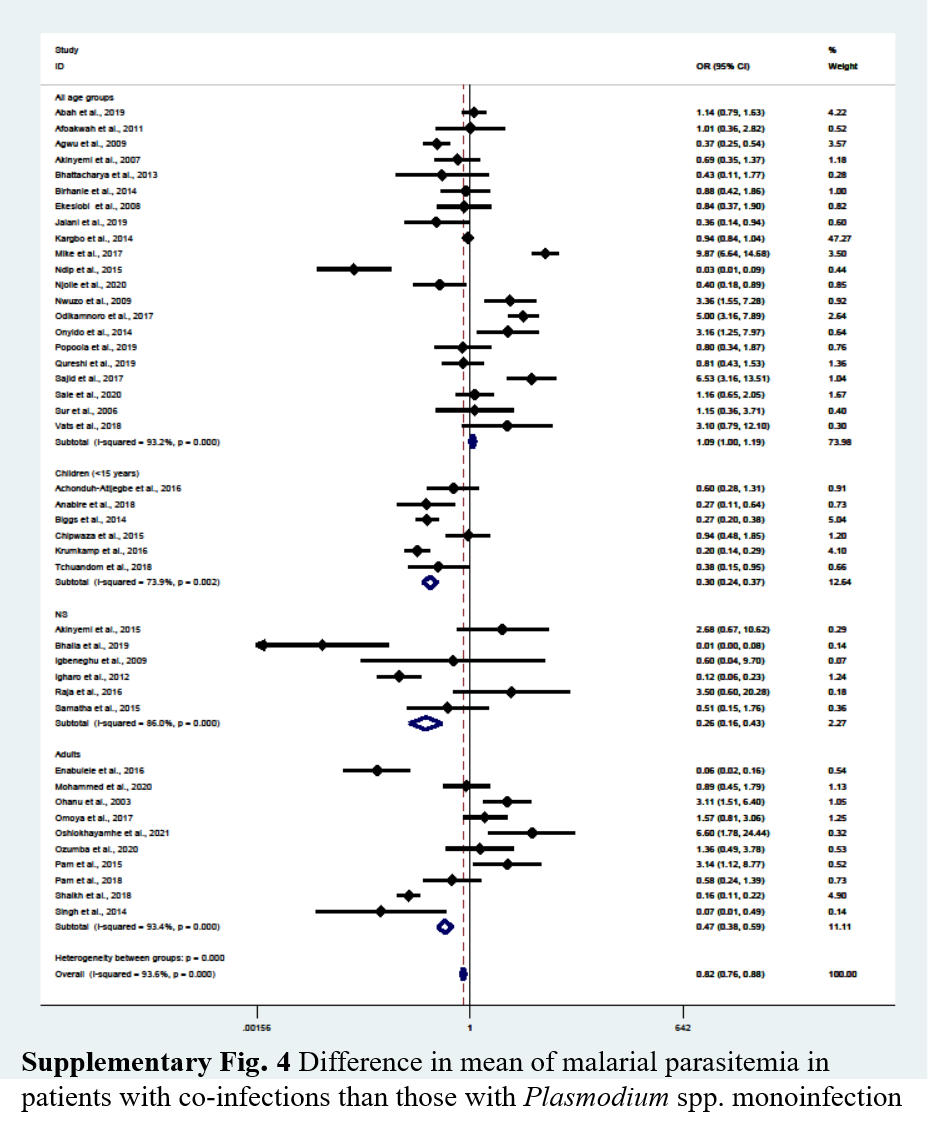

Supplement: Supplementary file 4 — Supplementary Figure S4. [file 41598_2021_611_MOESM4_ESM.png]

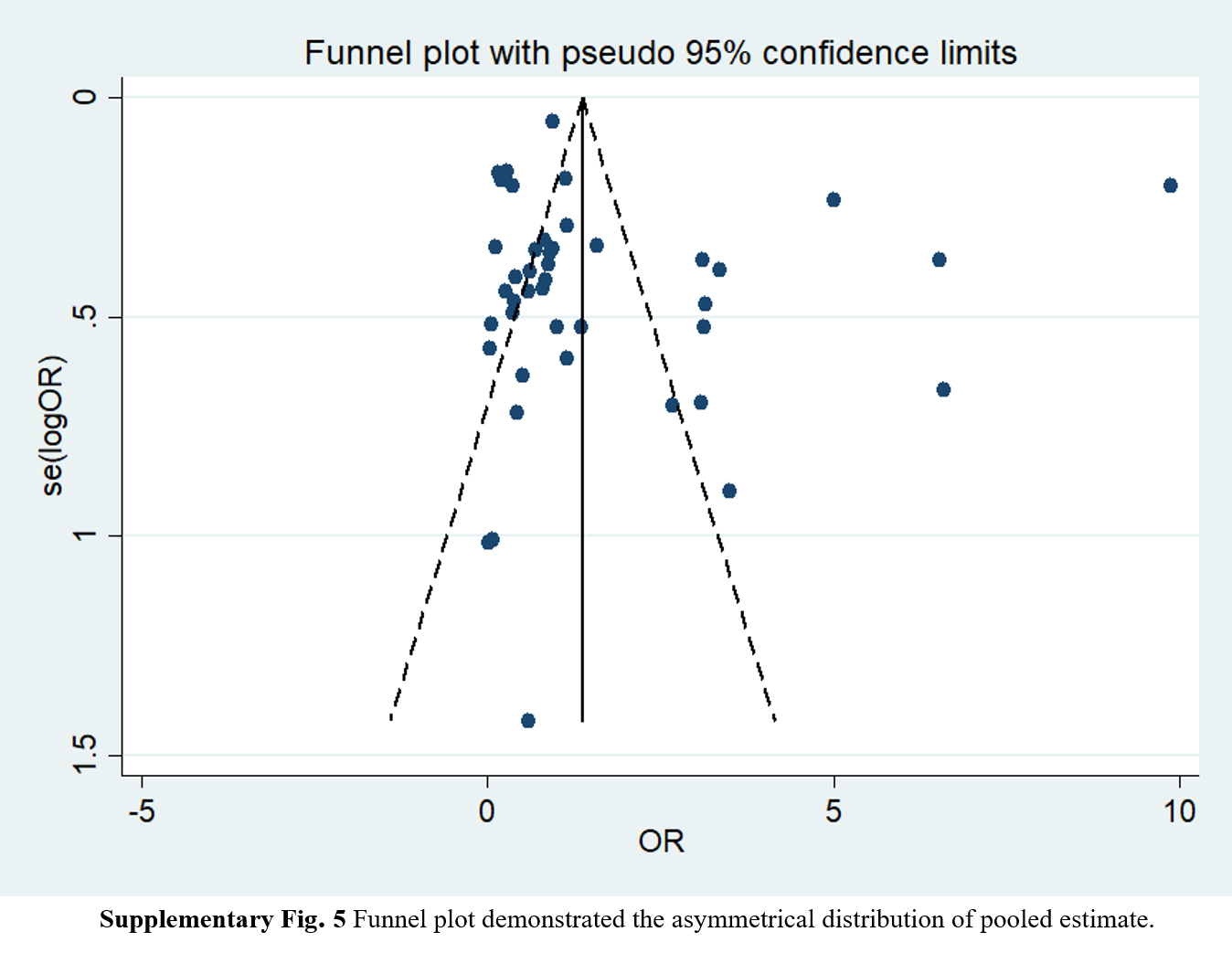

Supplement: Supplementary file 5 — Supplementary Figure S5. [file 41598_2021_611_MOESM5_ESM.png]

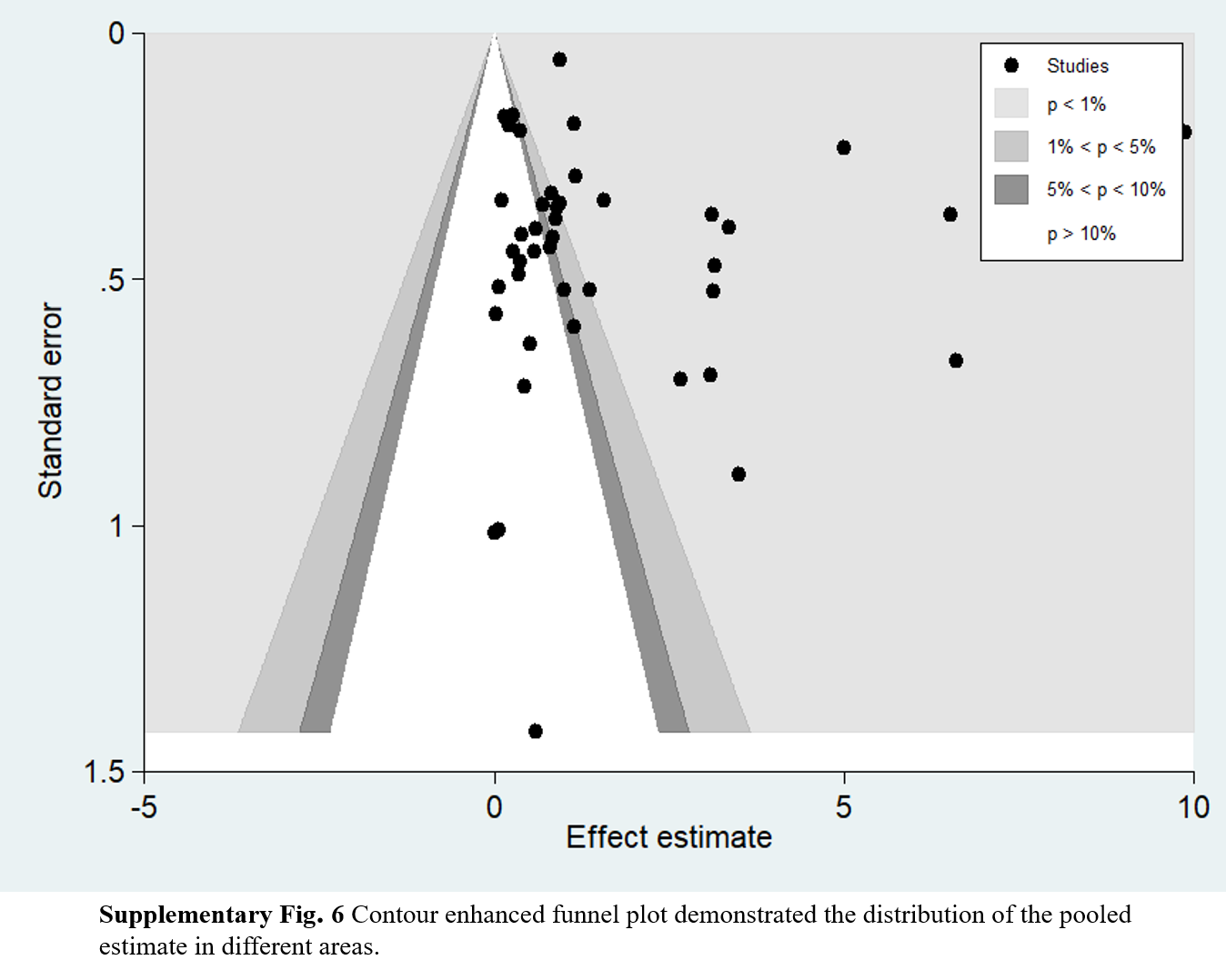

Supplement: Supplementary file 6 — Supplementary Figure S6. [file 41598_2021_611_MOESM6_ESM.png]
