# Supplementary material for: Prevalence, probability, and outcomes of typhoidal/non-typhoidal Salmonella and malaria co-infection among febrile patients: a systematic review and meta-analysis
Source: Sci Rep. 2021 Nov 8;11:21889. doi: 10.1038/s41598-021-00611-0 (PMC8576030; doi:10.1038/s41598-021-00611-0)
Supplement: Supplementary file 7 — Supplementary Table S1. [file 41598_2021_611_MOESM7_ESM.docx]

**Prevalence, probability, and outcomes of typhoidal/non-typhoidal *Salmonella* and malaria co-infection among febrile patients: a systematic review and meta-analysis**

Polrat Wilairatana^1^, Wanida Mala^2^, Wiyada Kwanhian Klangbud^2^, Kwuntida Uthaisar Kotepui^2^, Pongruj Rattaprasert^3^, Manas Kotepui^2*^

^1^Department of Clinical Tropical Medicine, Faculty of Tropical Medicine, Mahidol University, Bangkok, Thailand

^2^Medical Technology, School of Allied Health Sciences, Walailak University, Tha Sala, Nakhon Si Thammarat, Thailand

^3^Department of Protozoology, Faculty of Tropical Medicine, Mahidol University, Bangkok, Thailand

**Short title:** Prevalence of typhoidal/NTS and malaria co-infections

**^*^Corresponding author**

Manas Kotepui; [manas.ko@wu.ac.th](mailto:manas.ko@wu.ac.th), Tel.: +66954392469

Polrat Wilairatana; [polrat.wil@mahidol.ac.th](mailto:polrat.wil@mahidol.ac.th)

Wanida Mala; [wanida.ma@wu.ac.th](mailto:wanida.ma@wu.ac.th)

Wiyada Kwanhian Klangbud; [kwiyada@wu.ac.th](mailto:kwiyada@wu.ac.th)

Kwuntida Uthaisar Kotepui; [kwuntida.ut@wu.ac.th](mailto:kwuntida.ut@wu.ac.th)

Pongruj Rattaprasert; [pongruj.rat@mahidol.ac.th](mailto:pongruj.rat@mahidol.ac.th)

**Table S1. Search term**

| **Databases** | **Search terms** | **Date** |
| --- | --- | --- |
| MEDLINE | (malaria OR plasmodium) AND (salmonella OR typhoid OR "'enteric fever" OR typhus) AND (co-infection OR coinfection OR concurrent OR co-infected OR coinfected OR mixed OR polymicrobial)  Search results: 168 | 27 April 2021 |
| Scopus | (malaria OR plasmodium) AND (salmonella OR typhoid OR "'enteric fever" OR typhus) AND (co-infection OR coinfection OR concurrent OR co-infected OR coinfected OR mixed OR polymicrobial)  Search option: Title, abstract, keywords  Search results: 234 | 27 April 2021 |
| ISI Web of Science | (malaria OR plasmodium) AND (salmonella OR typhoid OR "'enteric fever" OR typhus) AND (co-infection OR coinfection OR concurrent OR co-infected OR coinfected OR mixed OR polymicrobial)  Search option: All fields  Search results: 148 | 27 April 2021 |
