# Supplementary material for: Prevalence, probability, and outcomes of typhoidal/non-typhoidal Salmonella and malaria co-infection among febrile patients: a systematic review and meta-analysis
Source: Sci Rep. 2021 Nov 8;11:21889. doi: 10.1038/s41598-021-00611-0 (PMC8576030; doi:10.1038/s41598-021-00611-0)
Supplement: Supplementary file 8 — Supplementary Table S2. [file 41598_2021_611_MOESM8_ESM.docx]

**Prevalence, probability, and outcomes of typhoidal/non-typhoidal *Salmonella* and malaria co-infection among febrile patients: a systematic review and meta-analysis**

Polrat Wilairatana^1^, Wanida Mala^2^, Wiyada Kwanhian Klangbud^2^, Kwuntida Uthaisar Kotepui^2^, Pongruj Rattaprasert^3^, Manas Kotepui^2*^

^1^Department of Clinical Tropical Medicine, Faculty of Tropical Medicine, Mahidol University, Bangkok, Thailand

^2^Medical Technology, School of Allied Health Sciences, Walailak University, Tha Sala, Nakhon Si Thammarat, Thailand

^3^Department of Protozoology, Faculty of Tropical Medicine, Mahidol University, Bangkok, Thailand

**Short title:** Prevalence of typhoidal/NTS and malaria co-infections

**^*^Corresponding author**

Manas Kotepui; [manas.ko@wu.ac.th](mailto:manas.ko@wu.ac.th), Tel.: +66954392469

Polrat Wilairatana; [polrat.wil@mahidol.ac.th](mailto:polrat.wil@mahidol.ac.th)

Wanida Mala; [wanida.ma@wu.ac.th](mailto:wanida.ma@wu.ac.th)

Wiyada Kwanhian Klangbud; [kwiyada@wu.ac.th](mailto:kwiyada@wu.ac.th)

Kwuntida Uthaisar Kotepui; [kwuntida.ut@wu.ac.th](mailto:kwuntida.ut@wu.ac.th)

Pongruj Rattaprasert; [pongruj.rat@mahidol.ac.th](mailto:pongruj.rat@mahidol.ac.th)

**Supplementary Table S2** Risk of bias among the included studies.

| No. | Authors | Eligibility criteria | Study subjects and the setting | Exposure measured in a valid and reliable way 'gold standard' | A specified diagnosis or definition | Confounding factors | Dealing with confounding factors | Outcomes measured in a valid and reliable way | Appropriate statistical analysis | Scores (8) | Risk of bias (high, moderate, low) |
| --- | --- | --- | --- | --- | --- | --- | --- | --- | --- | --- | --- |
| 1 | Abah et al., 2019 | Yes | Yes | No | Yes | No | NA | Yes | Yes | 6 | Moderate |
| 2 | Achonduh-Atijegbe et al., 2016 | Yes | Yes | No | Yes | No | NA | Yes | Yes | 6 | Moderate |
| 3 | Afoakwah et al., 2011 | Yes | No | No | Yes | No | NA | Yes | Yes | 5 | Moderate |
| 4 | Agwu et al., 2009 | Yes | Yes | No | Yes | No | NA | Yes | Yes | 6 | Moderate |
| 5 | Akinyemi et al., 2007 | Yes | Yes | Yes | Yes | No | NA | Yes | Yes | 7 | Low |
| 6 | Akinyemi et al., 2015 | Yes | No | No | Yes | No | NA | Yes | Yes | 6 | Moderate |
| 7 | Alhassan et al., 2012 | Yes | No | Yes | Yes | No | NA | Yes | Yes | 6 | Moderate |
| 8 | Ali et al., 2020 | Yes | Yes | No | Yes | No | NA | Yes | Yes | 6 | Moderate |
| 9 | Ammah et al., 1999 | Yes | Yes | No | Yes | No | NA | Yes | Yes | 6 | Moderate |
| 10 | Anabire et al., 2018 | Yes | Yes | No | Yes | No | NA | Yes | Yes | 6 | Moderate |
| 11 | Anjorin et al., 2020 | Yes | Yes | No | Yes | Yes | No | Yes | Yes | 5 | Moderate |
| 12 | Aung et al., 2018 | Yes | Yes | Yes | Yes | No | NA | Yes | Yes | 7 | Low |
| 13 | Bassat et al. 2009 | Yes | Yes | Yes | Yes | No | NA | Yes | Yes | 7 | Low |
| 14 | Berkley et al., 1999 | Yes | Yes | Yes | Yes | No | NA | Yes | Yes | 7 | Low |
| 15 | Bhalla et al., 2019 | Yes | No | No | Yes | Yes | No | Yes | Yes | 4 | Moderate |
| 16 | Bhattacharya et al., 2013 | Yes | Yes | Yes | Yes | No | NA | Yes | Yes | 7 | Low |
| 17 | Biggs et al., 2014 | Yes | Yes | Yes | Yes | No | NA | Yes | Yes | 7 | Low |
| 18 | Birhanie et al., 2014 | Yes | Yes | No | Yes | No | NA | Yes | Yes | 6 | Moderate |
| 19 | Brent et al., 2006 | Yes | Yes | No | Yes | No | NA | Yes | Yes | 6 | Moderate |
| 20 | Bronzan et al., 2007 | Yes | Yes | Yes | Yes | No | NA | Yes | Yes | 7 | Low |
| 21 | Chipwaza et al., 2015 | Yes | Yes | No | Yes | No | NA | Yes | Yes | 6 | Moderate |
| 22 | Chukwuma et al., 2014 | Yes | Yes | No | Yes | No | NA | Yes | Yes | 6 | Moderate |
| 23 | Edet et al., 2016 | Yes | Yes | Yes | Yes | No | NA | Yes | Yes | 7 | Low |
| 24 | Ekesiobi et al., 2008 | Yes | No | No | Yes | No | NA | Yes | Yes | 5 | Moderate |
| 25 | Enabulele et al., 2016 | Yes | No | Yes | Yes | No | NA | Yes | Yes | 6 | Moderate |
| 26 | Evans et al., 2004 | Yes | No | Yes | Yes | No | NA | Yes | Yes | 6 | Moderate |
| 27 | Eze et al., 2011 | Yes | No | No | Yes | No | NA | Yes | Yes | 5 | Moderate |
| 28 | Falay et al., 2016 | Yes | Yes | Yes | Yes | No | NA | Yes | Yes | 7 | Low |
| 29 | Graham et al., 2000 | Yes | Yes | Yes | Yes | No | NA | Yes | Yes | 7 | Low |
| 30 | Ibrahim et al., 2019 | Yes | Yes | No | Yes | No | NA | Yes | Yes | 6 | Moderate |
| 31 | Igbeneghu et al., 2009 | Yes | No | Yes | Yes | No | NA | Yes | Yes | 6 | Moderate |
| 32 | Igharo et al., 2012 | Yes | No | No | Yes | No | NA | Yes | Yes | 5 | Moderate |
| 33 | Jalani et al., 2019 | Yes | Yes | No | Yes | No | NA | Yes | Yes | 6 | Low |
| 34 | Kargbo et al., 2014 | Yes | Yes | No | Yes | No | NA | Yes | Yes | 6 | Moderate |
| 35 | Katiyar et al., 2020 | Yes | Yes | No | Yes | No | NA | Yes | Yes | 6 | Moderate |
| 36 | Krumkamp et al., 2016 | Yes | Yes | Yes | Yes | No | NA | Yes | Yes | 7 | Low |
| 37 | Mabey et al., 1987 | Yes | Yes | Yes | Yes | No | NA | Yes | Yes | 7 | Low |
| 38 | Maltha el al., 2014 | Yes | Yes | Yes | Yes | No | NA | Yes | Yes | 7 | Low |
| 39 | Mbuh et al., 2003 | Yes | Yes | Yes | Yes | No | NA | Yes | Yes | 7 | Low |
| 40 | Mike et al., 2017 | Yes | Yes | No | Yes | No | NA | Yes | Yes | 6 | Moderate |
| 41 | Mohammed et al., 2020 | Yes | Yes | No | Yes | No | NA | Yes | Yes | 6 | Moderate |
| 42 | Mourembou et al., 2016 | Yes | No | No | Yes | No | NA | Yes | Yes | 5 | Moderate |
| 43 | Mtove et al., 2010 | Yes | Yes | Yes | Yes | No | NA | Yes | Yes | 7 | Low |
| 44 | Ndip et al., 2015 | Yes | Yes | No | Yes | No | NA | Yes | Yes | 6 | Moderate |
| 45 | Nielsen et al., 2015 | Yes | Yes | Yes | Yes | No | NA | Yes | Yes | 7 | Low |
| 46 | Njolle et al., 2020 | Yes | Yes | No | Yes | No | NA | Yes | Yes | 6 | Moderate |
| 47 | Nwabueze et al., 2016 | Yes | No | No | Yes | No | NA | Yes | Yes | 5 | Moderate |
| 48 | Nwuzo et al., 2009 | Yes | Yes | No | Yes | No | NA | Yes | Yes | 6 | Moderate |
| 49 | Nyein et al., 2015 | Yes | Yes | Yes | Yes | No | NA | Yes | Yes | 7 | Low |
| 50 | Odikamnoro et al., 2017 | Yes | No | No | Yes | No | NA | Yes | Yes | 5 | Moderate |
| 51 | Ohanu et al., 2003 | Yes | Yes | Yes | Yes | No | NA | Yes | Yes | 7 | Low |
| 52 | Omoya et al., 2017 | Yes | Yes | No | Yes | No | NA | Yes | Yes | 6 | Moderate |
| 53 | Onyido et al., 2014 | Yes | Yes | No | Yes | No | NA | Yes | Yes | 6 | Moderate |
| 54 | Orok et al., 2016 | Yes | Yes | Yes | Yes | No | NA | Yes | Yes | 7 | Low |
| 55 | Oshiokhayamhe et al., 2021 | Yes | No | No | Yes | No | NA | Yes | Yes | 5 | Moderate |
| 56 | Oundo et al., 2002 | Yes | Yes | Yes | Yes | No | NA | Yes | Yes | 7 | Low |
| 57 | Ozumba et al., 2020 | Yes | Yes | No | Yes | No | NA | Yes | Yes | 6 | Moderate |
| 58 | Pam et al., 2015 | Yes | Yes | No | Yes | No | NA | Yes | Yes | 6 | Moderate |
| 59 | Pam et al., 2018 | Yes | Yes | No | Yes | No | NA | Yes | Yes | 6 | Moderate |
| 60 | Park et al., 2016 | Yes | Yes | Yes | Yes | No | NA | Yes | Yes | 7 | Low |
| 61 | Phu et al., 2020 | Yes | Yes | Yes | Yes | No | NA | Yes | Yes | 7 | Low |
| 62 | Popoola et al., 2019 | Yes | Yes | No | Yes | No | NA | Yes | Yes | 6 | Moderate |
| 63 | Qureshi et al., 2019 | Yes | Yes | No | Yes | No | NA | Yes | Yes | 6 | Moderate |
| 64 | Raja et al., 2016 | Yes | No | Yes | Yes | Yes | No | Yes | No | 4 | Moderate |
| 65 | Ramya et al., 2017 | Yes | No | No | Yes | Yes | No | Yes | Yes | 4 | Moderate |
| 66 | Sajid et al., 2017 | Yes | No | No | Yes | Yes | No | Yes | Yes | 4 | Moderate |
| 67 | Sale et al., 2020 | Yes | No | No | Yes | Yes | No | Yes | Yes | 4 | Moderate |
| 68 | Samatha et al., 2015 | Yes | No | Yes | Yes | Yes | No | Yes | Yes | 5 | Moderate |
| 69 | Sandlund et al., 2012 | Yes | Yes | Yes | Yes | No | NA | Yes | Yes | 7 | Low |
| 70 | Shaikh et al., 2018 | Yes | Yes | No | Yes | No | NA | Yes | Yes | 6 | Moderate |
| 71 | Sharma et al., 2016 | Yes | Yes | Yes | Yes | No | NA | Yes | Yes | 7 | Low |
| 72 | Singh et al., 2014 | Yes | Yes | Yes | Yes | No | NA | Yes | Yes | 7 | Low |
| 73 | Snehanshu et al., 2014 | Yes | Yes | Yes | Yes | No | NA | Yes | Yes | 7 | Low |
| 74 | Sur et al., 2006 | Yes | Yes | Yes | Yes | No | NA | Yes | Yes | 7 | Low |
| 75 | Tabu et al., 2012 | Yes | No | Yes | Yes | No | NA | Yes | Yes | 6 | Moderate |
| 76 | Tchuandom et al., 2018 | Yes | Yes | No | Yes | No | NA | Yes | Yes | 6 | Moderate |
| 77 | Ukaegbu et al., 2014 | Yes | No | No | Yes | Yes | No | Yes | Yes | 4 | Moderate |
| 78 | Vats et al., 2018 | Yes | No | Yes | Yes | Yes | No | Yes | Yes | 5 | Moderate |
| 79 | Verma et al., 2014 | Yes | No | Yes | Yes | No | NA | Yes | Yes | 6 | Moderate |
| 80 | Walsh et al., 2000 | Yes | Yes | Yes | Yes | No | NA | Yes | Yes | 7 | Low |
| 81 | Were et al., 2011 | Yes | Yes | Yes | Yes | No | NA | Yes | Yes | 7 | Low |

NA, Not Applicable
